# Genomic and evolutionary aspects of chloroplast tRNA in monocot plants

**DOI:** 10.1186/s12870-018-1625-6

**Published:** 2019-01-22

**Authors:** Tapan Kumar Mohanta, Abdul Latif Khan, Abeer Hashem, Elsayed Fathi Abd_ Allah, Dhananjay Yadav, Ahmed Al-Harrasi

**Affiliations:** 1 Natural and Medical Sciences Research Center, University of Nizwa, 616 Nizwa, Oman; 2 Botany and Microbiology Department, College of Science, King Saud University, Riyadh, 11451 Saudi Arabia; 3 Mycology and Plant Disease Survey Department, Plant Pathology Research Institute, Agriculture Research Center, Giza, Egypt; 4 Plant Production Department, College of Food and Agriculture Science, King Saud University, Riyadh, 11451 Saudi Arabia; 5 Department of Medical Biotechnology, Yeungnam University, Gyeongsan, 38541 Republic of Korea

**Keywords:** tRNA, Chloroplast, Anti-codon, Evolution, Transition, Transversion, Phylogenetics

## Abstract

**Background:**

Chloroplasts are one of the most indispensable organelles that make life forms on the earth possible by their capacity to photosynthesize. These organelles possess a circular genome with a number of coding genes responsible for self-regulation. tRNAs are an important evolutionary-conserved gene family that are responsible for protein translation. However, within the chloroplast genome, tRNA machinery are poorly understood.

**Results:**

In the present study, the chloroplast genome of six monocot plants, *Oryza nivara* (NC_005973), *Oryza sativa* (NC_001320), *Sachharum officinarum* (NC_006084), *Sorghum bicolor* (NC_008602), *Triticum aestivum* (NC_002762), and *Zea mays* (NC_001666) were downloaded and analyzed to identify tRNA sequences. Further analysis of the tRNA sequences in the chloroplast genomes of the monocot plants resulted in the identification of several novel features. The length of tRNAs in the chloroplast genome of the monocot plants ranged from 59 to 155 nucleotides. Pair-wise sequence alignment revealed the presence of a conserved A-C-x-U-A-x-U-A-x-U-x_5_-U-A-A nucleotide consensus sequence. In addition, the tRNAs in chloroplast genomes of the monocot plants also contain 21–28 anti-codons against 61 sense codons in the genome. They also contain a group I intron and a C-A-U anti-codon for tRNA^Ile^, which is a common anti-codon of tRNA^Met^. Evolutionary analysis indicates that tRNAs in the chloroplast genome have evolved from multiple common ancestors, and tRNA^Met^ appears to be the ancestral tRNA that underwent duplication and diversification to give rise to other tRNAs.

**Conclusion:**

The results obtained from the study of chloroplast tRNA will greatly help to increase our understanding of tRNA biology at a new level. Functional studies of the reported novel aspects of the chloroplast tRNA of the monocot plants will greatly help to decipher their roles in diverse cellular processes.

**Electronic supplementary material:**

The online version of this article (10.1186/s12870-018-1625-6) contains supplementary material, which is available to authorized users.

## Background

Chloroplasts are multi-copy cellular organelle [1] which are responsible for photosynthesis and carbohydrate metabolism in photoautotrophic plants which regulate our biosphere [2, 3]. They are an active metabolic center, and are responsible for sustaining the life on earth by converting solar energy into carbohydrates through the process of photosynthesis [4–6]. In addition to the major process of photosynthesis, chloroplasts also play an important role in various other molecular processes; including the synthesis of nucleotides, amino acids, fatty acids, vitamins, phytohormones, and several other metabolites [7–12]. Furthermore, they also contribute to the assimilation of nitrogen and sulphur [13–15]. In plants, these metabolites have been shown to play a critical role in the regulation of the physiology, growth, and development; as well as stress response. Therefore, chloroplasts can be regarded as the “metabolic center” of cellular reactions. Evolutionary studies indicate that chloroplasts have arisen from a cyanobacterial ancestor through internalization within a eukaryotic cell and have maintained an independent genome inside the plant cell [16–20]. The chloroplast genome (cpDNA) is a double stranded circular molecule containing tRNA, rRNA, and a number of protein coding genes [21]. The majority of the protein coding genes are associated with photosynthesis and bioenergetics [22, 23]. The chloroplast genome contains two large 6–76 Kb inverted repeats (IRs) that are divided into a large single copy (LSC) and small single copy region (SSC) [24–26]. The chloroplast genome is non-recombinant and inherited uniparentally through maternal inheritance [27, 28]. Therefore, the chloroplast genome is an excellent tool for genomic and evolutionary studies. It is very difficult, however, to detect polymorphisms in cpDNA due to a low level of substitutions [29, 30]. Recently, the advances in high-throughput genome sequencing technology have enabled rapid progress in the sequencing and analysis of chloroplast genomes. Specifically, these technological gains have enabled us to obtain and analyze the complete chloroplast genomes of several plants to better understand their molecular and genomic characteristics.

Since chloroplasts encode a complete and independent genome, it is important to study the chloroplast genomes; especially chloroplast tRNAs which are responsible for protein translation. Since the chloroplast genome is involved in the synthesis of nucleotides, amino acids and proteins, it is important to understand its organization to determine how these processes are regulated within the chloroplast genome. Protein translation within the chloroplasts is regulated by tRNA and other associated genes. Thus, detailed analyses of chloroplast tRNAs can provide insight into the genomics and evolution of cyanobacterial tRNAs. In relative comparison to eudicots, the monocot genome is more conserved than the eudicots genome, and they have evolved from the eudicot lineage [31–33]. In addition, several of the important agronomic crops species are monocots. Therefore, in the present study, we considered to study the chloroplast genome of six monocot plants to better understand the genomic and evolutionary characteristics of the chloroplast tRNA that can enable functional studies for the future.

tRNAs are one of the most important and versatile molecules responsible for sustaining and maintaining the protein translation machinery. They are characterized by the presence of a clover leaf-like structure as proposed by Robert Holley [34]. This structure contains features such as an acceptor arm, D-arm, D-loop, anti-codon arm, anti-codon loop, variable arm, pseudouridine arm, and pseudouridine loop. The tRNAs are encoded within the nuclear genome and in the genome of sub-cellular organelles, including plastids and mitochondria. Over the years, detailed studies pertaining to the characterization of nuclear tRNA have gained considerable attention [35–37]. Structure and function of tRNAs and tRNA genes of chloroplast genome was previously described by Mareachal-Drouard et al., (1991) [38]. However, due to the lack of complete genome sequences of chloroplast genome, the study lacked the complete genomic details of tRNAs of plastid genome. Therefore, we attempted to understand the detailed genomic and molecular aspects of chloroplast tRNA in plants. Considering the conserved evolutionary lineages of monocots, six economically important monocots were investigated and reported within this study.

## Results

### Genomic of chloroplast tRNA

**Fig. 1:**
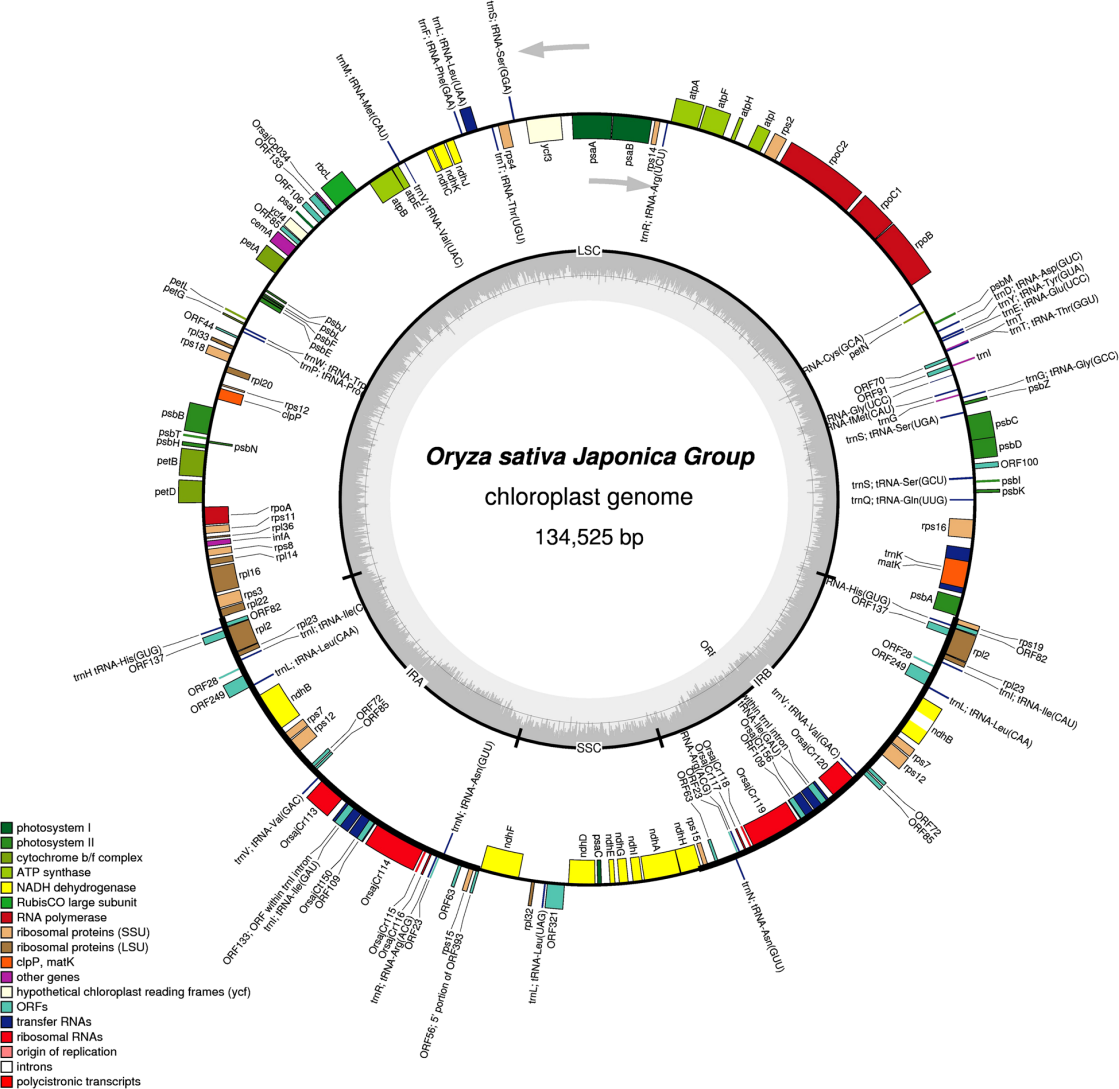
Organization of chloroplast genome. The genome map shows the presence of tRNAs and other genes

The whole chloroplast genome sequence of six monocot plants, *Oryza nivara* (NC_005973), *Oryza sativa* (NC_001320), *Sachharum officinarum* (NC_006084), *Sorghum bicolor* (NC_008602), *Triticum aestivum* (NC_002762), and *Zea mays* (NC_001666), were downloaded from the National Center of Biotechnology Information (NCBI) database. Subsequently, the sequences were annotated to identify the genomic tRNA sequences in these genomes (Fig. 1). The obtained genomic tRNA sequences were further analyzed using the tRNAscan-Se server to confirm their identity as tRNAs. Results indicated that *O. nivara*, *O. sativa*, *S. officinarum*, *S. bicolor*, *T. aestivum*, and *Z. mays* encode 38, 35, 37, 29, 39, and 39 tRNAs, respectively (Table 1). The length of the chloroplast tRNAs ranged from 59 nt [tRNA^Thr^ GGU, *Sorghum bicolor*, (20385)] to 155 nt [tRNA^Lys^ NNN, *T. aestivum*, (4982_TraeCt095)]. tRNA^Gly^ UCC of *O. nivara* (6129) was found to contain only 65 nt, whereas tRNA^Gln^ UUG of *T. aestivum* (4985), and tRNA^Leu^ UAG of *T. aestivum* (5086_TraeCt128) contained 118 nt and 100 nt, respectively. In the tRNA, tRNA^Gln^ UUG (4985_TraeCt096), the tRNA begins at 46 nt and in tRNA^Leu^ UAG (5086_TraeCt128), it begins at 21 nt. Pairwise sequence alignment of 5′ nucleotide sequence of these two tRNAs revealed a 22.2% similarity (55.6% gaps) and the presence of a conserved A-C-x-U-A-x-U-A-x-U-x_5_-U-A-A consensus sequence. On average, chloroplast tRNAs in the examined monocot plants contain 76 nucleotides. tRNA^Cys^, tRNA^Asn^, tRNA^Ala^, tRNA^Asp^, tRNA^Phe^, and tRNA^Trp^ were found to contain 71, 72, 73, 74, 73, and 74 nucleotides, respectively. All of the sequences of the tRNA^Leu^ and tRNA^Ser^ were found to contain 80 nt or more. tRNA^Lys^ was found to be absent from the chloroplast genome of *O. sativa* and *S. bicolor* (Table 1). Additionally, tRNA^Ala^ and tRNA^Ile^ were also found to be absent in *S. bicolor* (Table 1).

**Table 1:** Distribution of tRNA isotypes in the chloroplast genome of the monocot plants

tRNA isotypes	No. of tRNAs					
	*O. nivara*	*O. sativa*	*S. officinarum*	*S. bicolor*	*T. aestivum*	*Z. mays*
Alanine	2	2	2	0	2	2
Glycine	2	1	2	1	2	2
Proline	1	1	1	1	1	1
Threonine	2	2	2	1	2	3
Valine	3	2	3	2	3	3
Serine	3	3	3	3	3	3
Arginine	3	3	3	3	3	3
Leucine	4	4	4	3	4	4
Phenylalanine	1	1	1	1	1	1
Asparagine	2	2	1	2	2	2
Lysine	1	0	1	0	1	1
Aspartate	1	1	1	1	1	1
Glutamate	1	1	1	1	1	1
Histidine	2	2	2	2	2	2
Glutamine	1	1	1	1	1	1
Isoleucine	4	4	4	4	4	4
Methionine	2	2	2	2	3	2
Tyrosine	1	1	1	1	1	1
Cysteine	1	1	1	1	1	1
Tryptophan	1	1	1	1	1	1
Selenocysteine	0	0	0	0	0	0
Suppressor	0	0	0	0	0	0
Total	38	35	37	31	39	39

### Chloroplast tRNAs of monocot plant encodes 21–28 anti-codons only

The chloroplast genomes of the investigated monocot plants, however, were found to encode only 21–28 anti-codons (Table 2). The chloroplast genome of *O. nivara*, *O. sativa*, *S. officinarum*, *S. bicolor*, *T. aestivum*, and *Z. mays* encoded 28, 25, 28, 21, 28, and 28 anti-codons, respectively (Table 2). The most common anti-codons found in the tRNA of chloroplast genome were UGC (tRNA^Ala^), GCC (tRNA^Gly^), UCC (tRNA^Gly^), UGG (tRNA^Pro^), GGU (tRNA^Thr^), UGU (tRNA^Thr^), GAC (tRNA^Val^), UAC (tRNA^Val^), GGA (tRNA^Ser^), UGA (tRNA^Ser^), GCU (tRNA^Ser^), ACG (tRNA^Arg^), UCU (tRNA^Arg^), UAG (tRNA^Leu^), CAA (tRNA^Leu^), UAA (tRNA^Leu^), GAA (tRNA^Phe^), GUU (tRNA^Asn^), UUU (tRNA^Lys^), GUC (tRNA^Asp^), UUC (tRNA^Glu^), GUG (tRNA^His^), UUG (tRNA^Gln^), CAU (tRNA^Ile^), GAU (tRNA^Ile^) CAU (tRNA^Met^), GUA (tRNA^Tyr^), GCA (tRNA^Cys^), and CCA (tRNA^Trp^) (Table 2). The UCC (tRNA^Gly^), and UAC (tRNA^Val^) anti-codons present in the genome of *O. nivara* were missing in the chloroplast genome of the related species, *O. sativa* (Table 2). Similarly, the anti-codons UCC (tRNA^Gly^), and UAC (tRNA^Val^) present in the genome of *O. nivara*, *S. officinarum*, *T. aestivum,* and *Z. mays* were found to be absent in the genome of *S. bicolor* (Table 2). In addition, the anti-codons GGU (tRNA^Thr^) and UAA (tRNA^Leu^) were also not present in *S. bicolor*; whereas, they were found in *O. nivara*, *O. sativa*, *S. officinarum*, *T. aestivum* and *Z. mays*. Outside of the above mentioned 28 anti-codons, the rest of the 33 anti-codons were not found in any of the tRNAs of the investigated monocot chloroplast genomes (Table 2).

**Table 2:** Distribution of anti-codons in the chloroplast genome of the monocot plants

tRNA Isotypes	Isoacceptors					
*Oryza nivara* (29)						
Alanine	AGC	GGC	CGC	UGC (2)		
Glycine	ACC	GCC (1)	CCC	UCC (1)		
Proline	AGG	GGG	CGG	UGG (1)		
Threonine	AGU	GGU (1)	CGU	UGU (1)		
Valine	AAC	GAC (2)	CAC	UAC (1)		
Serine	AGA	GGA (1)	CGA	UGA (1)	ACU	GCU (1)
Arginine	ACG (2)	GCG	CCG	UCG	CCU	UCU (1)
Leucine	AAG	GAG	CAG	UAG (1)	CAA (2)	UAA (1)
Phenylalanine	AAA	GAA (1)				
Asparagine	AUU	GUU (2)				
Lysine	CUU	UUU (1)				
Aspartate	AUC	GUC (1)				
Glutamate	CUC	UUC (1)				
Histidine	AUG	GUG (2)				
Glutamine	CUG	UUG (1)				
Isoleucine	AAU	GAU (2)	CAU (2)			
Methionine	CAU (4)					
Tyrosine	AUA	GUA (1)				
Cysteine	ACA	GCA (1)				
Tryptophan	CCA (1)					
Supressor	CUA	UUA				
Selenocysteine	UCA					
*Oryza sativa* (26)						
Alanine	AGC	GGC	CGC	UGC (2)		
Glycine	ACC	GCC (1)	CCC	UCC		
Proline	AGG	GGG	CGG	UGG (1)		
Threonine	AGU	GGU (1)	CGU	UGU (1)		
Valine	AAC	GAC (2)	CAC	UAC		
Serine	AGA	GGA (1)	CGA	UGA (1)	ACU	GCU (1)
Arginine	ACG (2)	GCG	CCG	UCG	CCU	UCU (1)
Leucine	AAG	GAG	CAG	UAG (1)	CAA (2)	UAA (1)
Phenylalanine	AAA	GAA (1)				
Asparagine	AUU	GUU (2)				
Lysine	CUU	UUU				
Aspartate	AUC	GUC (1)				
Glutamate	CUC	UUC (1)				
Histidine	AUG	GUG (2)				
Glutamine	CUG	UUG (1)				
Isoleucine	AAU	GAU (2)	CAU (2)			
Methionine	CAU (4)					
Tyrosine	AUA	GUA (1)				
Cysteine	ACA	GCA (1)				
Tryptophan	CCA (1)					
Supressor	CUA	UUA				
Selenocysteine	UCA					
*Saccharum officinarum* (29)						
Alanine	AGC	GGC	CGC	UGC (2)		
Glycine	ACC	GCC (1)	CCC	UCC (1)		
Proline	AGG	GGG	CGG	UGG (1)		
Threonine	AGU	GGU (1)	CGU	UGU (1)		
Valine	AAC	GAC (2)	CAC	UAC (1)		
Serine	AGA	GGA (1)	CGA	UGA (1)	ACU	GCU (1)
Arginine	ACG (2)	GCG	CCG	UCG	CCU	UCU (1)
Leucine	AAG	GAG	CAG	UAG (1)	CAA (2)	UAA (1)
Phenylalanine	AAA	GAA (1)				
Asparagine	AUU	GUU (2)				
Lysine	CUU	UUU (1)				
Aspartate	AUC	GUC (1)				
Glutamate	CUC	UUC (1)				
Histidine	AUG	GUG (2)				
Glutamine	CUG	UUG (1)				
Isoleucine	AAU	GAU (2)	CAU (2)			
Methionine	CAU (4)					
Tyrosine	AUA	GUA (1)				
Cysteine	ACA	GCA (1)				
Tryptophan	CCA (1)					
Supressor	CUA	UUA				
Selenocysteine	UCA					
*Sorghum bicolor* (23)						
Alanine	AGC	GGC	CGC	UGC		
Glycine	ACC	GCC (1)	CCC	UCC		
Proline	AGG	GGG	CGG	UGG (1)		
Threonine	AGU	GGU	CGU	UGU (1)		
Valine	AAC	GAC (2)	CAC	UAC		
Serine	AGA	GGA (1)	CGA	UGA (1)	ACU	GCU (1)
Arginine	ACG (2)	GCG	CCG	UCG	CCU	UCU (1)
Leucine	AAG	GAG	CAG	UAG (1)	CAA (2)	UAA
Phenylalanine	AAA	GAA (1)				
Asparagine	AUU	GUU (2)				
Lysine	CUU	UUU				
Aspartate	AUC	GUC (1)				
Glutamate	CUC	UUC (1)				
Histidine	AUG	GUG (2)				
Glutamine	CUG	UUG (1)				
Isoleucine	AAU	GAU (2)	CAU (2)			
Methionine	CAU (4)					
Tyrosine	AUA	GUA (1)				
Cysteine	ACA	GCA (1)				
Tryptophan	CCA (1)					
Supressor	CUA	UUA				
Selenocysteine	UCA					
*Triticum aestivum* (28)						
Alanine	AGC	GGC	CGC	UGC (2)		
Glycine	ACC	GCC (1)	CCC	UCC (1)		
Proline	AGG	GGG	CGG	UGG (1)		
Threonine	AGU	GGU (1)	CGU	UGU (1)		
Valine	AAC	GAC (2)	CAC	UAC (1)		
Serine	AGA	GGA (1)	CGA	UGA (1)	ACU	GCU (1)
Arginine	ACG (2)	GCG	CCG	UCG	CCU	UCU (1)
Leucine	AAG	GAG	CAG	UAG (1)	CAA (2)	UAA (1)
Phenylalanine	AAA	GAA (1)				
Asparagine	AUU	GUU (2)				
Lysine	CUU	UUU (1)				
Aspartate	AUC	GUC (1)				
Glutamate	CUC	UUC (1)				
Histidine	AUG	GUG (1)				
Glutamine	CUG	UUG				
Isoleucine	AAU	GAU (2)	CAU (2)			
Methionine	CAU (5)					
Tyrosine	AUA	GUA (1)				
Cysteine	ACA	GCA (1)				
Tryptophan	CCA (1)					
Supressor	CUA	UUA				
Selenocysteine	UCA					
*Zea mays* (29)						
Alanine	AGC	GGC	CGC	UGC (2)		
Glycine	ACC	GCC (1)	CCC	UCC (1)		
Proline	AGG	GGG	CGG	UGG (1)		
Threonine	AGU	GGU (2)	CGU	UGU (1)		
Valine	AAC	GAC (2)	CAC	UAC (1)		
Serine	AGA	GGA (1)	CGA	UGA (1)	ACU	GCU (1)
Arginine	ACG (2)	GCG	CCG	UCG	CCU	UCU (1)
Leucine	AAG	GAG	CAG	UAG (1)	CAA (2)	UAA (1)
Phenylalanine	AAA	GAA (1)				
Asparagine	AUU	GUU (2)				
Lysine	CUU	UUU (1)				
Aspartate	AUC	GUC (1)				
Glutamate	CUC	UUC (1)				
Histidine	AUG	GUG (2)				
Glutamine	CUG	UUG (1)				
Isoleucine	AAU	GAU (2)	CAU (2)			
Methionine	CAU (2)					
Tyrosine	AUA	GUA (1)				
Cysteine	ACA	GCA (1)				
Tryptophan	CCA (1)					
Supressor	CUA	UUA				
Selenocysteine	UCA					

### Conservation of chloroplast tRNA sequences is family specific

Multiple sequence alignment analysis of all 20 tRNA gene family members of studied monocot species revealed small, highly conserved consensus sequences in the pseudouridine (Ψ) loop, but not in the other parts of the tRNA (Table 3). The Ψ-loop was found to possess a conserved U-U-C-x-A consensus nucleotide sequence (Table 3). The majority of the tRNAs contained a G nucleotide at the first position. tRNA^Val^, tRNA^Met^, and tRNA^Pro^, however, were found to possess an A nucleotide at the first position instead of a G (Table 3). tRNA^Gln^ and tRNA^Asn^ were found to possess a U nucleotide at the first position in the acceptor arm. Although no consensus sequence conservation was observed in the 5′-acceptor arm, the D-arm contained a conserved C nucleotide at the 4th position of the arm (13th position of the tRNA). In contrast, tRNA^Glu^, tRNA^Gly^, tRNA^Met^, tRNA^Ser^, tRNA^Tyr^, and all other tRNAs, possessed a C nucleotide at the 4th position of the D-arm. Nucleotide 7 to 16 of the canonical tRNA forms an A box, which has been reported to contain two conserved consensus sequences, ^7^GUGGCNNAGU^16^- and -GGU-AGNGC^15^ (− stands for gap & N stands for any nucleotide) [39]. Our analysis revealed that among the 20 tRNAs analyzed, only six of them possess a conserved G nucleotide at the 7th position (Table 3). The 7th position of the tRNA is instead occupied by an A, U, or C nucleotide (Table 3). The 14th position (1st nucleotide of D-loop) was found to be conserved in the majority of tRNA. Except for tRNA^Arg^, tRNA^Asn^, tRNA^Gly^, and tRNA^Met^, all other tRNAs were found to contain a conserved A nucleotide at the 14th position (Table 3). Similarly, the last nucleotide of the D-loop was found to be a conserved A nucleotide except tRNA^Tyr^ (Table 3). The consensus sequence ^52^GGUUCGANUCC^62^, which starts from the 52nd position and ends at the 62nd position of tRNA, forms a B box [40]. Our analysis indicates that the conservation of box A and B nucleotide sequences in tRNA occurs in a family-specific manner. The G-G nucleotide at the 52nd and 53rd position was found to be conserved in the majority of tRNAs, except for tRNA^Glu^, tRNA^Lys^, and tRNA^Val^; whereas, the nucleotide sequence U-U-C-x-A-x-U was found to conserved at the 54th, 55th, 56th, 58th, and 60th positions (Table 3). tRNA^Met^ was found to contain a conserved U-U-C-x-A-U-C consensus sequence at the 54th, 55th, 56th, 58th, 59th, and 60th positions, instead of the U-U-C-x-A-x-U consensus sequence (Table 3). Similarly, tRNA^Asp^ had a conserved U-U-C-G-A-G-C consensus sequence, while tRNA^Val^ contained U-U-C-G-A-x-x conserved nucleotides. No conserved nucleotides were found at the 59th and 60th positions of tRNA^Val^. The anti-codon loop at the 32nd and 33rd positions were found to contain conserved C-U or U-U nucleotides. tRNA^Gln^, tRNA^Gly^, tRNA^His^, tRNA^Pro^, and tRNA^Val^ contained conserved U-U nucleotides instead of the C-U nucleotides. In addition, in the majority of cases, the anti-codon loop at the 38th position had a conserved A nucleotide. tRNA^Gln^, tRNA^Pro^, and tRNA^Val^, however, possessed a conserved U nucleotide at the 38th position instead of nucleotide A (Table 3). The chloroplast genome encodes a predefined C-C-A tail in the gene of the tRNA. When the tRNA gene is transcribed, a C-C-A tail is included. The present study found that tRNA^Ala^, tRNA^Arg^, tRNA^Ile^, tRNA^Lys^, and tRNA^Tyr^ contain C-C-A nucleotides in their 3′-end. A few of the encoded tRNA^Leu^ genes in the monocot chloroplast genomes also contain C-C-A tail in the 3′-end, however, the remaining tRNAs do not possess a C-C-A consensus sequence at their 3′-end.

**Table 3:** Multiple sequence alignment and the presence of isotype specific conserved nucleotide consensus sequence in chloroplast tRNA of monocot plant. Asteriks indicates no conserved sequence

tRNA Isotypes	AC arm	D-arm	D-loop	ANC arm	ANC loop	Variable region	Ψ-arm	Ψ-loop
Alanine	G-G-G-G-A-U-A	G-C-U-C	A-G-U-U-G-G-U-A	C-C-G-C-U	C-U-U-G-C-A-A	A-U-G-U-C	A-G-C-G-G	U-U-C-G-A-G-U
Arginine	G-x-G	G-x-U-C	G-x_3_-A	********	C-U-x_3_-A-A	U-G	G-G	U-U-C-x-A-x-U
Asparagine	U-C-C-U-C-A-G	G-C-U-C	G-A-U-G-G-U-A	G-U-C-G-C	C-U-G-U-U-A-A	U-G-G-U-C	G-U-A-G-G	U-U-C-G-A-A-U
Aspartate	G-G-G-A-U-U-G	G-U-U-C	A-A-U-U-G-G-U-C-A	C-C-G-C-C	C-U-G-U-C-A-A	A-A-G-C-U	G-C-G-G-G	U-U-C-G-A-G-C
Cysteine	G-G-C-G-G-C-A	G-C-C	A-A-G-x-G-G-U-A-A	G-G-G-G-A	C-U-G-C-A-A-A	U-A-x-C	C-C-C-A-G	U-U-C-A-A-A-U
Glutamate	G-C-C-C-C-x-A	G-U-C-U	A-G-U-G-G-U-U-C-A	U-C-U-C-U	C-U-U-U-C-A-A	C-A-G-C	G-G-G-G-A	U-U-C-G-A-C-U
Glutamine	U-G-G-G-G-C-G	G-C-C	A-A-G-U-G-G-U-A-A	G-C-G-G-G	U-U-U-U-G-G-U	U-A-C-U-C	G-G-A-G-G	U-U-C-G-A-A-U
Glycine	G-C-G-A-G-C-G	G-U-U	C-A-x-U-G-G-U-A-A	U-C-U-C-C	U-U-G-C-C-A-A	A-G-A-U-A	C-C-G-G-G	U-U-C-G-A-U-U
Histidine	G-C-G-G-A-U-G	G-C-C	A-A-G-U-G-G-A-U-C-A-A	G-U-G-G-A	U-U-G-U-G-A-A	C-A-U-G-C	G-C-G-G-G	U-U-C-A-A-U-U
Isoleucine	G-G-G-C-U-A-U	G-C-U-C	A-G-U-G-G-U-A	C-G-C-C-C	C-U-G-A-U-A-A	A-G-G-U-C	U-C-U-G-G	U-U-C-A-A-G-U
Leucine	G-x_5_-A	G-x-G	A-A-U-x-G-U-A-G-A	*******	C-U-x-A-x_2_-A	G-x_1–5_-U-x_2_-A-x_3–5_-U	G-G	U-U-C-x-A-G-U
Lysine	G-G-G-U-U-G-C	A-C-U-C	A-A-U-G-G-U-A	U-C-G-G	C-U-U-U-U-A-A	C-U-A	******	U-U-C-G-A-G-U
Methionine	G-C	*****	U-x-G-x-U-A	*******	x-U-C-A-U-A-x	U	G-G	U-U-C-x-A-U-C
Phenylalanine	G-U-C-A-G-G-A	G-C-U-C	A-G-U-U-G-G-U-A	G-A-G-G-A	C-U-G-A-A-A-A	G-U-G-U-C	A-C-C-A-G	U-U-C-A-A-A-U
Proline	A-G-G-G-A-U-G	G-C-G-C	A-G-C-U-U-G-G-U-A	U-U-U-G-U	U-U-U-G-G-G-U	A-U-G-U-C	A-C-A-G-G	U-U-C-A-A-A-U
Serine	G-G-A-G-A-G-A	G-C-x-G	A-G-x-G-G-x_3_-A	G	C/U-U-G/U-x_2_-A	U-x-U-A-x_4_-U-x_5–6_-U-A-x-C	G-A-G-G-G	U-U-C-G-A-A-U
Threonine	G-C-C-C-x_2_-U	C-U-C	A-G-x-G-G-U-x-A	G-C	x-U-x-G-U-A-A	G-U-C	A-U-C-G-G	U-U-C-A-A-A-U
Tryptophan	G-C-G-C-U-C-U	G-U-U-C	A-G-U-U-x-G-G-U-A	C-G-G-G-U	C-U-C-C-A-A-A	A-U-G-U-C	G-U-A-G-G	U-U-C-A-A-A-U
Tyrosine	G-G-G-U-C-G-A	C-C-C-G	A-G-x-G-G-U-U-A-U	A-C-G-G-A	C-U-G-U-A-A-A	U-G-A-C-x-A-U-x_2_-G-U-C-U-A-C	G-C-U-G-G	U-U-C-A-A-A-U
Valine	A-G-G-G-x-U-A	C-U-C	A-G-x_0–2_-C-G-G-U-A	C-x-C	U-U-x-A-C-x-U	A-x-G-U-C	C-x-G	U-U-C-G-A-x-x

### Nucleotide variation in the arms and loops of tRNA

In the present study, the acceptor arm of chloroplast tRNA was revealed to contain 1–7 nucleotides. Among the 213 tRNA sequences representing six species of monocot plants, only two were found to contain one nucleotide, one had five nucleotides, and one contained six nucleotides; while the rest of the 209 (98.12%) tRNAs had seven nucleotides. The D-arm was found to contain 3 and 4 nucleotides and none of the tRNAs possessed less than three or more than four nucleotides in the D-arm. A total of 73 (34.25%) were had three nucleotides, while 140 (65.73%) were contained four nucleotides. The D-loop, that forms a part of the A box, had seven to eleven nucleotides. Among the 213 tRNAs, 45 (21.12%) of the D-loops contain seven, 38 (17.84%) contain 8, 75 (35.21%) contain nine, 22 (10.32%) contain 10, and 33 (15.49%) contain 11 nucleotides. The anti-codon arm of the chloroplast tRNAs had 4–5 nucleotides. Among the 213 tRNAs, 23 (10.79%) of the anti-codon arms contain four nucleotides, while 190 (89.20%) contain five nucleotides (Additional file 1: Table S1). All of the tRNAs, except for one, had seven nucleotides in the anti-codon loop. tRNA 6160_OrniCt018 of *O. nivara* contained nine nucleotides instead of seven (Additional file 1: Table S1). The variable loop was found to possess a diverse number of nucleotides with different tRNAs having 4 (9.38%), 5 (59.62%), 6 (3.75%), 7 (5.63%), 11 (2.34%), 12 (0.46%), 13 (6.1%), 14 (0.46%), 15 (1.87%), 16 (2.34%), 18 (2.34%), or 19 (5.63%) nucleotides. None of the chloroplast tRNAs were found to possess 8, 9, 10, 17, 20 or more nucleotides in the variable loop (Additional file 1: Table S1). tRNA^Leu^, tRNA^Ser^, and tRNA^Tyr^ had 10 or more nucleotides, respectively, whereas the other tRNAs possessed less than 10 nucleotides in the variable loop (Additional file 1: Table S1). Among the 213 examined tRNA sequences, only three tRNA^Gly^ genes had four nucleotides in the Ψ-arm, while the remaining tRNA sequences had five nucleotides. Similarly, the Ψ-loop region in all of the 213 tRNAs possessed seven nucleotides. Our study found 7 bp in the acceptor arm and 3–4 bp in the D-arm and considerable variation was observed in the other parts. The anti-codon arm was found to possess 4–5 bp, and the anti-codon loop 7 or 9 nucleotides. The number of nucleotides making up the variable loop ranged from 4 to 19 and none of the tRNAs had more than 19 nucleotides in the variable loop. Similar to the previous report, the Ψ-arm possessed 4–5 nucleotides.

### Chloroplast tRNA contain group I intron

**Fig. 2:**
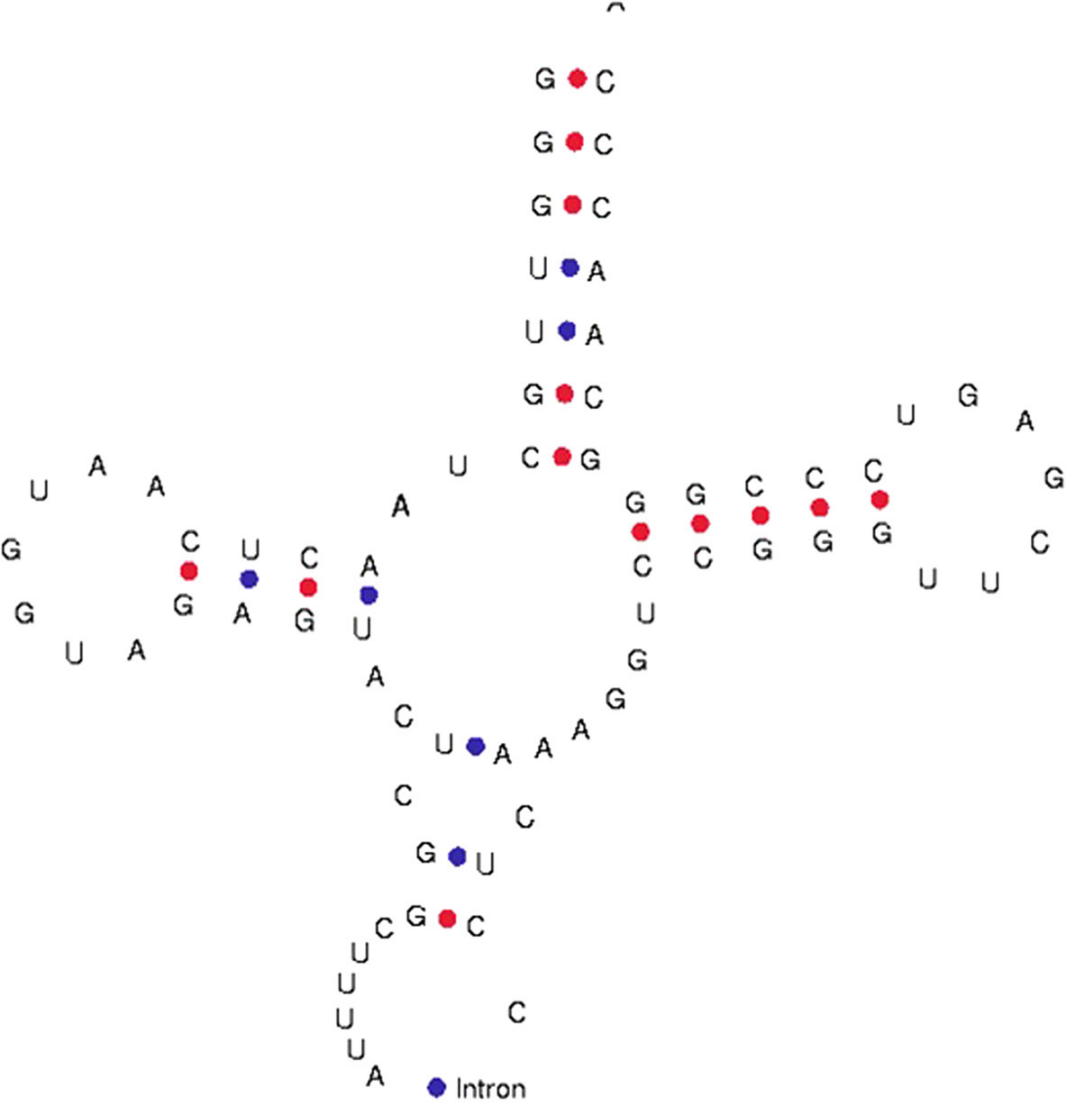
Presence of group I intron in chloroplast tRNA. The intron was found to locate in the anti-codon loop of the tRNA

**Fig. 3:**
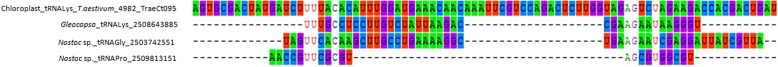
Multiple sequence alignment of group I intron of chloroplast tRNA with the group I intron of cyanobacteria. Multiple sequence alignment shows the presence of U-U-×7-U-U-×35-A-G-x2-U

**Fig. 4:**
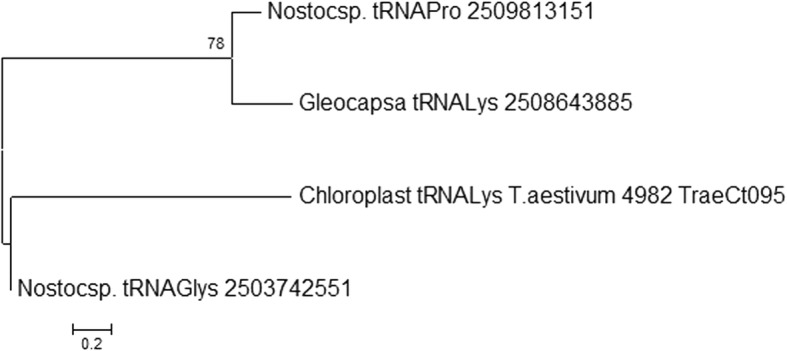
Phylogenetic tree of group I intron of chloroplast tRNA. The group I intron of chloroplast tRNA grouped with the group I intron of *Nostoc* sp. explaining cyanobacterial origin of group I intron of chloroplast

In our study, however, chloroplast tRNA was found to contain introns. tRNA^Lys^ of *T. aestivum* (4982_TraeCt095) was found to contain a group I intron located in the anti-codon loop region of tRNA^Lys^ (Fig. 2). The intron was 84 nucleotides in length and began at nucleotide 37 and ended at nucleotide 120 of the tRNA^Lys^ gene. The group I introns of chloroplast tRNA contain conserved U-U-x_2_-C and A-G-x_2_-U consensus sequences (Fig. 3). A phylogenetic tree was constructed to elucidate the evolution of the group I intron. The phylogenetic analysis indicated that the group I intron of chloroplast tRNA grouped with the group I intron of cyanobacteria (Fig. 4).

### Chloroplast tRNA encodes putative novel tRNAs

**Fig. 5:**
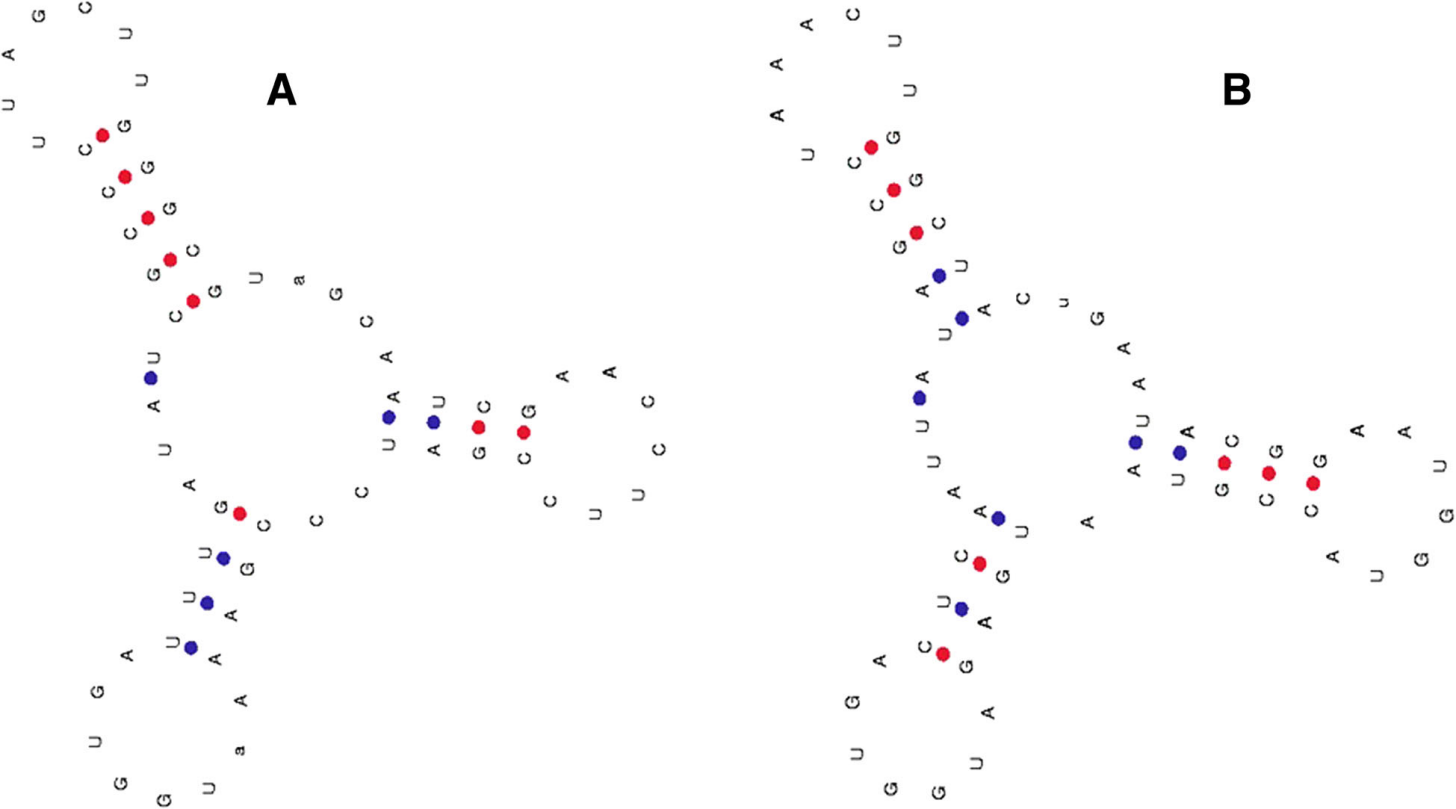
Structure of novel chloroplast tRNA lacking acceptor arm. **a** tRNA^Gly^ of *O. nivara* (id: 6129) and **b** tRNA^Thr^ of *S. bicolor* (id: 20385) do not contain acceptor arm

**Fig. 6:**
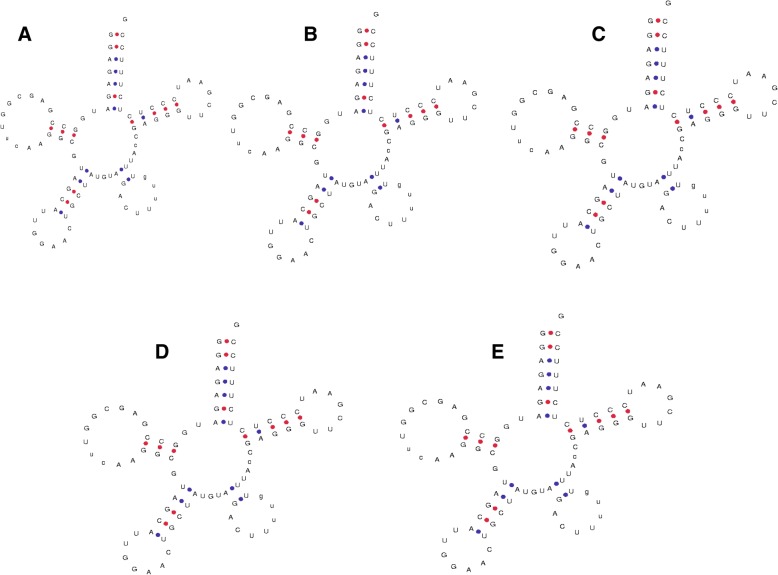
Structure of chloroplast tRNA showing the presence of a seven nucleotides loop structure similar to the anti-codon loop. Proximal presence of loop-like structure in the variable loop region of tRNA most probably mimics the anti-codon loop. tRNA^Ser^ of (**a**) *O. nivara* (id: 6152), (**b**) *O. sativa* (id: 3720), (**c**) *S. bicolor* (id: 20407), (**d**) *S. officinarum*, (id: 6593) and (**e**) *T. aestivum* (id: 5020) was found to contain a seven nucleotides loop structure in the variable loop region

**Fig. 7:**
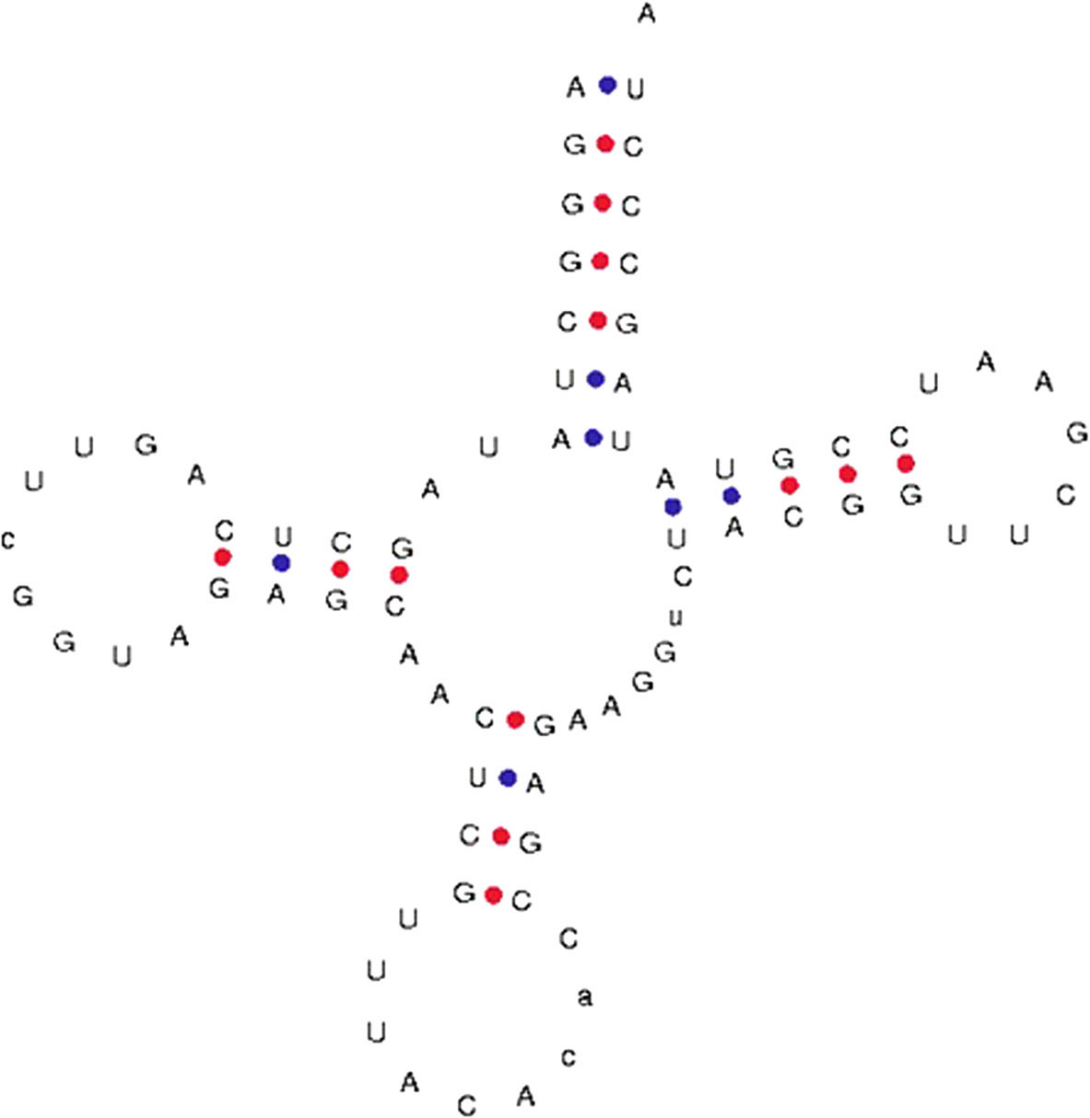
Novel anti-codon loop of tRNA. The anti-codon loop of tRNAVal of *O. sativa* (id: 6160) contains nine nucleotides in the anti-codon loop instead of seven

In the present study, a few putative novel tRNAs were found to be encoded by the chloroplast genome (Fig. 5). tRNA^Gly^ (UCC) of *O. nivara* (6129_OrniCt007, ΔG = − 18.10), and tRNA^Thr^ (GGU) of *S. bicolor* (20,385_trnM-CAU SobiCt011, ΔG = 14.7) did not contain an acceptor arm at the 5′-end (Fig. 5). Additionally, a few tRNA^Ser^ in *O. nivara* (6152_OrniCt014, ΔG = − 34.13), *O. sativa* (3720_OrsajCt137, ΔG = − 34.13), *S. bicolor* (20,407_trnS-GGA SobiCt019, ΔG = − 34.13), *S. officinarum* (6593), and *T. aestivum* (5020_TraeCt112, ΔG = − 34.13) were found to contain a seven-nucleotide loop structure in the variable loop region, similar to the anti-codon loop of tRNA (Fig. 6). All of the loop structures comprising the variable loop region were found to be composed of A-C-U-U-U-U-G nucleotides. The tRNA^Val^ of *O. nivara* (6160_OrniCt018, ΔG = − 25.20) was found to contain only four nucleotides in the anti-codon arm and nine nucleotides in the anti-codon loop (Fig. 7). Many similar tRNA structures have been found in the genomic tRNA of cyanobacteria, as well as plants (unpublished data).

#### C-A-U anti-codon codes for tRNA^Ile^ in chloroplast tRNAs

The C-A-U anti-codon is a characteristic feature of tRNA^Met^ and has only one iso-acceptor. In addition to the presence of a C-A-U anti-codon in tRNA^Met^, we also found that the tRNA^Ile^ of chloroplast tRNA also encodes a C-A-U anti-codon. The tRNA^Ile^ in *O. nivara* (6206_OrniCp049, 6270_OrniCt035), *O. sativa* (3774_OrsajCt146, 3828_OrsajCt160), *S. officinarum* (officinarum_6644, officinarum_6710), *S. bicolor* (20,460, 20,502), *T. aestivum* (5069, 5108), and *Z. mays* (2069_trnI ZemaCt144, 2131_trnI ZemaCt154) chloroplast genomes encode a C-A-U anti-codon. To our knowledge, this may be the first report to document the presence of a C-A-U anti-codon in chloroplast tRNA^Ile^.

## Chloroplast tRNAs have evolved from multiple common ancestors

**Fig. 8:**
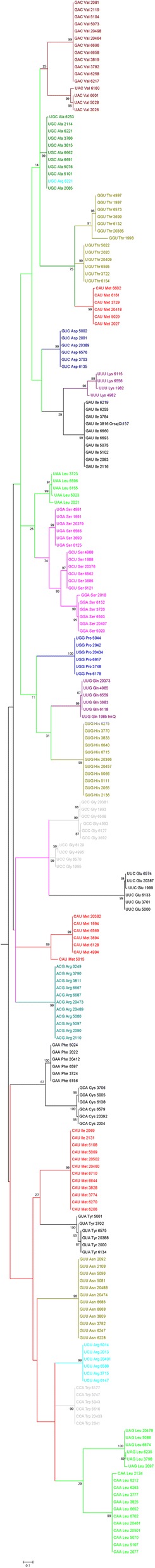
Phylogenetic tree of chloroplast tRNAs. Phylogenetic analysis revealed polyphyletic origin of chloroplast tRNAs. tRNAMet underwent vivid duplication and diversification to give rise other tRNAs in chloroplast. Phylogenetic tree was constructed by MEGA7 using maximum likelihood analysis and 1000 boot strap replicates

A phylogenetic tree was constructed using the tRNA sequences in the chloroplast genomes of all of the examined monocot plants. A phylogenetic analysis revealed the presence of two major clusters that consist of 30 groups. Cluster I contain tRNA^Val^, tRNA^Ala^, tRNA^Arg^, tRNA^Thr^, tRNA^Met^, tRNA^Asp^, tRNA^Lys^, tRNA^Ile^, tRNA^Leu^, tRNA^Ser^, tRNA^Pro^, tRNA^Gln^, tRNA^His^, tRNA^Gly^, tRNA^Glu^, and tRNA^Arg^. Cluster II contains tRNA^Phe^, tRNA^Cys^, tRNA^Ile^, tRNA^Met^, tRNA^Tyr^, tRNA^Asn^, tRNA^Arg^, tRNA^Trp^, and tRNA^Leu^. There are 21 groups in cluster I and 9 groups in cluster II (Fig. 8). In cluster I, tRNA^Arg^ is grouped twice; once with tRNA^Ala^ and once near to tRNA^Met^. Similarly, tRNA^Met^ is also grouped twice; once near to the group containing tRNA^Thr^ and once near the group containing tRNA^Arg^ (Fig. 8). tRNA^Arg^, tRNA^Ile^, tRNA^Leu^, and tRNA^Met^ present in cluster I are also found in cluster II of the phylogenetic tree. The tRNAs with the anti-codon G-A-C and U-A-C of tRNA^Val^, G-G-U and U-G-U of tRNA^Thr^, U-G-A, G-C-U, and G-G-A of tRNA^Ser^, G-C-C and U-C-C of tRNA^Gly^, U-A-A, U-A-G, and C-A-A of tRNA^Leu^; C-A-U of tRNA^Ile^, U-G-C, U-C-U, and A-C-G of tRNA^Arg^, all grouped separately (Fig. 8). tRNA^Trp^ (CCA) is closely grouped with tRNA^Arg^ (UCU) in cluster II, suggesting the evolution of tRNA^Trp^ from tRNA^Arg^ (Fig. 8). Similarly, tRNA^Tyr^ (GUA) is closely grouped with tRNA^Met^ (CAU) and tRNA^Ile^ (CAU), suggesting the evolution of tRNA^Tyr^ (GUA) and tRNA^Ile^ (CAU) from tRNA^Met^ (CAU). The grouping of tRNA^Met^ (CAU) with tRNA^Ile^ (CAU), and their similar anti-codon nucleotides, strongly suggests that tRNA^Ile^ evolved directly from tRNA^Met^. In addition, the close grouping of tRNA^Met^ (CAU) with tRNA^Arg^ (ACG) further suggests that tRNA^Arg^ has evolved from tRNA^Met^ as well. The grouping of tRNA^Glu^ (UUC) with tRNA^Gly^ (GCC), tRNA^His^ (GUG) with tRNA^Gln^ (UUG), and tRNA^Pro^ (UGG) suggests that these tRNAs may have evolved from a common ancestor or by a gene duplication event. tRNA^Ser^ (GGA, GCU, UGA) grouped with tRNA^Leu^ (UAA); which suggests that tRNA^Ser^ evolved from tRNA^Leu^. Notably, tRNA^Leu^ contains a C-A-A anti-codon, while tRNA^Leu^_,_ which grouped with tRNA^Ser^, contains a U-A-A anti-codon. This suggests that tRNA^Leu^ (CAA) has undergone a base substitution to give rise to tRNA^Leu^ (UAA) and that further duplication and diversification resulted in tRNA^Ser^ (GGA, GCU, UGA). The grouping of tRNA^Ile^ (GAU), tRNA^Lys^ (UUU), and tRNA^Asp^ (GUC) together suggests their common evolutionary lineage. Further, grouping of tRNA^Met^ with tRNA^Thr^ (UGU and GGU) suggests that tRNA^Thr^ (UGU and GGU) evolved from tRNA^Met^. Similarly, the close phylogenetic relationship of tRNA^Met^ with tRNA^Ala^ and tRNA^Val^ in cluster I indicates that tRNA^Ala^ and tRNA^Val^ also evolved from tRNA^Met^. A disparity index test of substitution pattern homogeneity was conducted using Monte Carlo replications to determine if all of the substitutions and the rate of substitution of the nucleotides are homogenous. Results indicated that the null hypothesis was rejected for tRNA^Arg^, tRNA^Gln^, tRNA^Ala^, tRNA^Met^, tRNA^Thr^, and tRNA^Val^; suggesting that the rate of substitution of nucleotides in these groups is homogenous. Outside of these six tRNA isotypes, 14 did not show pattern homogeneity, and hence, the substitution of nucleotides and evolution of tRNA^Gly^, tRNA^Pro^, tRNA^Ser^, tRNA^Leu^, tRNA^Phe^, tRNA^Asn^, tRNA^Lys^, tRNA^Asp^, tRNA^Glu^, tRNA^His^, tRNA^Ile^, tRNA^Tyr^, tRNA^Cys^, and tRNA^Trp^ are not homogenous. To better understand the relationship of chloroplast tRNAs with the Archaea, we incorporated tRNA two Archaea species and the tRNA sequences of three cyanobacterial species were used as ingroups. The complementary DNA sequences of two *Arabidopsis thaliana* NAC transcription factors (AtNAC1 and AtNAC2) were used as out groups (Additional file 2: Figure S1). A phylogenetic analysis showed some overlapping relationship of Archaea tRNAs with the chloroplast tRNA. However, chloroplast tRNAs were much closer to cyanobacterial tRNA compared to the Archaea.

### The rate of transition and transversion is Isoacceptor specific

tRNAs are evolutionarily conserved molecules and the possibility of undergoing major transition or transversion events is very minimum. The rate of transition (8.33) and transversion (8.34) of tRNA^Ala^, tRNA^Asn^, tRNA^Asp^, tRNA^His^, tRNA^Phe^, and tRNA^Pro^ are almost equal. This indicates that, although the rate of transversion is slightly higher than the rate of transition, these tRNAs have evolved at almost an equal rate with respect to transition and transversion (Table 4). Additionally, the rate of transition (25.00) and transversion (0.00) of tRNA^Cys^, tRNA^Gln^, tRNA^Trp^, and tRNA^Tyr^ were also similar to each other (Table). Notably, however, tRNA^Cys^, tRNA^Gln^, tRNA^Trp^, and tRNA^Tyr^ in the chloroplast genome of monocot plants have undergone a high rate of transition but have not undergone any transversion. In contrast, the rate of transversion in tRNA^Ile^ (8.60), tRNA^Lys^ (10.09), tRNA^Ser^ (9.15), was found to be higher relative to the rate of transition for tRNA^Ile^ (7.80), tRNA^Lys^ (4.82), and tRNA^Ser^ (6.70), respectively (Table 4). A higher transition rate was also observed in tRNA^Arg^ (12.40), tRNA^Glu^ (12.53), tRNA^Gly^ (17.39), tRNA^Leu^ (11.88), tRNA^Met^ (16.87), tRNA^Thr^, and tRNA^Val^ (Table 4). The highest rate of transition substitutions (25.00) was found in tRNA^Cys^, tRNA^Gln^, tRNA^Trp^, and tRNA^Tyr^. When all of the tRNAs are collectively examined, however, the average rate of transition (14.71) is greater than the average rate of transversion (5.15) (Table 4).

**Table 4:** Transition and transversion rate of the chloroplast tRNAs. Bold letter indicates transition

	A	U	C	G		A	U	C	G
Alanine					Lysine				
A	–	8.34	8.34	**8.33**	A	–	10.09	10.09	**4.82**
U	8.34	–	**8.33**	8.34	U	10.09	**–**	**4.82**	10.09
C	8.34	**8.33**	–	8.34	C	10.09	**4.82**	–	10.09
G	**8.33**	8.34	8.34	–	G	**4.82**	10.09	10.09	–
Arginine					Methionine				
A	–	6.3	6.3	**12.40**	A	–	4.06	4.06	**16.87**
U	6.30	–	**12.40**	6.30	U	4.06	–	**16.87**	4.06
C	6.30	**12.40**	–	6.30	C	4.06	**16.87**	–	4.06
G	**12.40**	6.30	6.30	–	G	**16.87**	4.06	4.06	–
Asparagine					Phenylalanine				
A	–	8.34	8.34	**8.33**	A	–	8.34	8.34	**8.33**
U	8.34	–	**8.33**	8.34	U	8.34	–	**8.33**	8.34
C	8.34	**8.33**	–	8.34	C	8.34	**8.33**	–	8.34
G	**8.33**	8.34	8.34	–	G	**8.33**	8.34	8.34	–
Aspartate					Proline				
A	–	8.34	8.34	**8.33**	A	–	8.34	8.34	**8.33**
U	8.34	–	**8.33**	8.34	U	8.34	–	**8.33**	8.34
C	8.34	**8.33**	–	8.34	C	8.34	**8.33**	–	8.34
G	**8.33**	8.34	8.34	–	G	**8.33**	8.34	8.34	–
Cysteine					Serine				
A	–	0.00	0.00	**25.00**	A	–	9.15	9.15	**6.70**
U	0.00	–	**25.00**	0.00	U	9.15	–	**6.70**	9.15
C	0.00	**25.00**	–	0.00	C	9.15	**6.70**	–	9.15
G	**25.00**	0.00	0.00	–	G	**6.70**	9.15	9.15	–
Glutamine					Threonine				
A	–	0.00	0.00	**25.00**	A	–	5.15	5.15	**14.70**
U	0.00	–	**25.00**	0.00	U	5.15	–	**14.70**	5.15
C	0.00	**25.00**	–	0.00	C	5.15	**14.70**	–	5.15
G	**25.00**	0.00	0.00	–	G	**14.70**	5.15	5.15	–
Glutamate					Tryptophan				
A	–	6.23	6.23	**12.53**	A	–	0.00	0.00	**25.00**
U	6.23	–	**12.53**	6.23	U	0.00	–	**25.00**	0.00
C	6.23	**12.53**	–	6.23	C	0.00	**25.00**	–	0.00
G	**12.53**	6.23	6.23	–	G	**25.00**	0.00	0.00	–
Glycine					Tyrosine				
A	–	3.80	3.80	**17.39**	A	–	0.00	0.00	**25.00**
U	3.80	–	**17.39**	3.80	U	0.00	–	**25.00**	0.00
C	3.80	**17.39**	–	3.80	C	0.00	**25.00**	–	0.00
G	**17.39**	3.80	3.80	–	G	**25.00**	0.00	0.00	–
Histidine					Valine				
A	–	8.34	8.34	**8.33**	A	–	5.45	5.45	**14.10**
U	8.34	–	**8.33**	8.34	U	5.45	–	**14.10**	5.45
C	8.34	**8.33**	–	8.34	C	5.45	**14.10**	–	5.45
G	**8.33**	8.34	8.34	–	G	**14.10**	5.45	5.45	–
Isoleucine					Overall				
A	–	8.60	8.60	**7.80**	A	–	5.15	5.15	**14.71**
U	8.60	–	**7.80**	8.60	U	5.15	–	**14.71**	5.15
C	8.60	**7.80**	–	8.60	C	5.15	**14.71**	–	5.15
G	**7.80**	8.60	8.60	–	G	**14.71**	5.15	5.15	–
Leucine									
A	–	6.56	6.56	**11.88**					
U	6.56	–	**11.88**	6.56					
C	6.56	**11.88**	–	6.56					
G	**11.88**	6.56	6.56	–					

### Duplication of chloroplast tRNA precedes over deletion

**Fig. 9:**
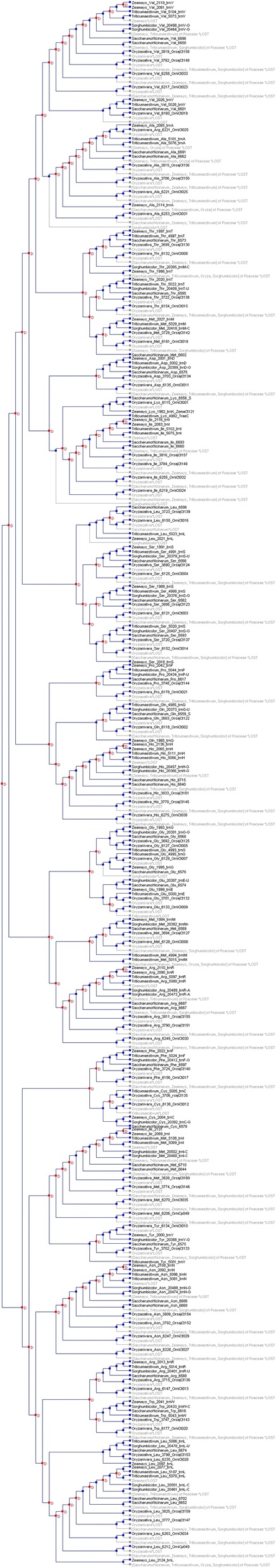
Duplication and loss events of chloroplast tRNAs. Analysis shows that chloroplast tRNAs underwent vivid gene loss during the course of evolution with subsequent diversification. Duplication loss study was conducted using Notung software

Plant genomes contain a greater abundance of duplicated genes and whole genome duplication events have occurred multiple times over the past 200 million years [41–44]. Given the cyanobacterial origin of the chloroplast genome, the rate of duplication and loss events could be different from genes within the nuclear-encoded genome. In the present study, duplication/loss analyses of chloroplast tRNA in monocot plants revealed that 101 genes experienced a duplication event and that 139 genes underwent losses; whereas, 80 genes underwent conditional duplication. The majority of chloroplast tRNAs underwent losses during the course of evolution. Although all of the tRNAs descended from the same lineage (monocot), the loss of genes was still greater than the duplicated genes (Fig. 9).

## Discussion

tRNAs are conserved family genes responsible for conducting protein translation event. Their presence in the chloroplast genome is supplementary to the genome to make it semi-autonomous. Multiple sequence alignment of chloroplast tRNAs revealed several basic conserved genomic features. A few tRNAs were found to contain extended nucleotide sequences at the 5′-end. However, the tRNAscan-SE server was not able to confirm if these nucleotide sequences of the 5′-end were introns. As a result, it is highly possible that these sequences can be introns of the tRNAs. A previous study reported the presence of a group I intron in cyanobacterial tRNA [45]. Given the origin of the chloroplast genome from a cyanobacterial lineage, it is reasonable to consider that these sequences are most likely introns of the chloroplast tRNAs [45]. Analysis of each tRNA sequence revealed tRNA^Leu^ and tRNA^Ser^ encoded for longest tRNA sequences. A previous study also reported the presence of 80 or more nucleotides in tRNA^Leu^ and tRNA^Ser^ of *Oryza sativa* [45]. This indicates that tRNA^Leu^ and tRNA^Ser^ encode longer tRNA sequences as compared to the others. This study also revealed the absence of tRNA^Lys^, tRNA^Ala^, and tRNA^Ile^ genes in the chloroplast genome of these monocot plants. The absence of important tRNA encoding genes in the chloroplast genome is quite intriguing and makes it important to understand how protein translation in these monocot plants is conducted in the absence of important tRNAs. Most likely, genomic tRNA compensate for the absence of plastidal tRNAs or it might be possible that other tRNAs from the organellar genome perform multiple functions to conduct protein translation. This is the first report regarding the absence of tRNA^Lys^, tRNA^Ala^, and tRNA^Ile^ in the chloroplast genome. In addition to the absence of tRNA^Lys^, tRNA^Ala^, and tRNA^Ile^, the chloroplast genome of monocot plants also lacks selenocystein, pyrrolysine and suppressor tRNA (Table 1). Our analysis also revealed that the monocot chloroplast genome contains the highest number genes encoding tRNA^Leu^ and tRNA^Met^; (4) followed by tRNA^Arg^, and tRNA^Ser^ (3). The universal genetic table contains 64 codons; of which, 61 are sense and 3 are anti-sense codons. Therefore, it is possible that there will be tRNAs with 61 unique anti-codons to code for 61 sense codons. Approximately 33 anti-codons were found to be absent from the tRNAs of chloroplast genome. However, the absence of UCC anti-codons of tRNA^Gly^ is compensated by the presence of GCC anti-codons of tRNA^Gly^, whereas the absence of anti-codon UAC of tRNA^Val^ is compensated by the presence of GAC anti-codons of tRNA^Val^. Similarly, the anti-codon GGU of tRNA^Thr^ is compensated by the presence of the UGG anti-codon of tRNA^Thr^ and the anti-codon UAA of tRNA^Leu^ is compensated by the presence of anti-codon UAG and CAA. The complete absence of a tRNA gene for tRNA^Lys^ (UUU, CUU) in *O. sativa* and *S. bicolor,* and tRNA^Ala^ (AGC, GGC, CGC, and UGC) is difficult to understand. Nevertheless, it can be speculated that the deficiency created by the absence of these tRNAs in the chloroplast genome might be compensated by genomic tRNAs or other tRNAs of chloroplast or nuclear origin. The anti-codon CAU is encoded by tRNA^Met^ and tRNA^fMet^. Our analysis indicated that chloroplast genome of the investigated monocot plants encodes tRNA^Met^ and tRNA^fMet^ as well. Previously, Howe (1985) and Hiratsuka et al., (1989) reported the presence of tRNA^fMet^ in chloroplast genome [46, 47]. All of the species were found to contain at least one tRNA^fMet^ and one tRNA^Met^. *O. nivara* (6128_OrniCt006), *O. sativa* (3694_OrsajCt127), *S. officinarum* (6569), *S. bicolor* (20382), *T. aestivum* (4994), and *Z. mays* (1994) each encode one tRNA^fMet^. In the prokaryotic genome, the initiation of protein translation is mediated by tRNA^fMet^, whereas subsequent addition of methionine to the polypeptide chain is mediated by tRNA^Met^ [48–50]. The presence of tRNA^Met^ and tRNA^fMet^ is a characteristic feature of prokaryotic and organellar genes [51] and the presence of tRNA^fMet^ in the chloroplast genome of monocot plants suggests its prokaryotic origin.

tRNAs are an evolutionarily conserved multigene family due to their functional similarities across many species. The nucleotide composition of a tRNA is responsible for maintaining the tertiary structure of the translated tRNA. Thus, the common conserved functions of tRNA should also be reflected in conserved coding sequences. A previous study reported the presence of a conserved nucleotide consensus sequence in tRNAs which was confined to the Ψ-loop only [45]. In our study, we found the presence of U-U-C-x-A nucleotide consensus sequence in the Ψ-loop. However, no conserved consensus sequences were found in other parts of the tRNAs. Instead, they were found to contain some conserved nucleotides. The nuclear encoded tRNA^Gln^ and tRNA^Asn^ contain a U nucleotide at the first position (Table 3) [45]. However, a multiple sequence alignment study indicated that the sequence conservation present in chloroplast tRNAs is family specific (Table 3). During protein translation, polymerase binds with the promotor of the tRNA which is known as A and B box. These two boxes contain conserved consensus sequences. Box A starts at the + 8 nucleotide of mature tRNA, whereas box B contains conserved ^52^GGUUCGANUCC^62^ nucleotides consensus that constitutes a part of the Ψ-arm and whole Ψ-loop. Box A of chloroplast tRNA was not so conserved, whereas box B was highly conserved. Boxes A and B are considered to be the intragenic transcription promotor signal sequence for RNA polymerase III [52]. The signal sequence for transcription activation is not conserved in a universal manner in the tRNAs of the chloroplast genome. The anti-codon loop was reported to be conserved at the 32nd position [52]. However, in the present study, conservation of nucleotides was found at the 32nd and 33rd positions in the majority of cases. In addition, several tRNA sequences were found to contain 3’-C-C-A tail. The addition of a C-C-A tail to the 3′-end of a tRNA is facilitated by a tRNA nucleotidlyltransferase. However, chloroplast genomes do not encode tRNA nucleotidyltransferases. Thus, adding a C-C-A tail to the 3′-end of the tRNA would be difficult in the absence of nucleotidyltransferases. The absence of a C-C-A tail at the 3′ end of the few tRNAs reflect their recent evolution as the majority of nuclear tRNAs lacked a 3’ C-C-A tail.

Given the cyanobacterial origin of the chloroplast genome, it should be prokaryotic in nature, and in general, should be intron free. However, we found the presence of group I introns in the chloroplast tRNAs. Previous studies have also reported the presence of intron in tRNA^Leu^ (UAA) and tRNA^fMet^ (UAC) of cyanobacterial tRNA [53, 54]. Additionally, a recent study conducted in our laboratory also reported the presence of introns in cyanobacterial tRNA^Arg^, tRNA^Gly^, and tRNA^Lys^ [45]. Although the presence of introns in the cyanobacterial genome has been reported by several studies, the present study appears to be the first to report the presence of introns in chloroplast tRNA. The group I introns lack significant sequence conservation, however, the present analysis indicated that they contain short conserved consensus sequences. The group I intron of chloroplast tRNA grouped with the group I intron of cyanobacteria (Fig. 4), thus providing additional evidence to suggest that they evolved from a common cyanobacterial lineage.

As proposed by Robert Holley [34], tRNAs are characterized by a cloverleaf-like structure, although a few tRNAs vary in their secondary structure [35]. tRNAs contains various arms and loops that function in protein translation. Each arm and loop have their own unique nucleotide composition. A previous study reported that the acceptor arm contains seven base pairs 7 bp, the D-stem 3–4 bp, the D-loop 4–12 nucleotides, the anti-codon arm 5 bp, the anti-codon loop 7 nucleotides, the variable region 4–23 nucleotides, the Ψ-arm 5 bp, and the Ψ-loop seven nucleotides [37]. The previous report, along with the present study, suggests that significant variation exists in arms and loops of chloroplast tRNAs. The acceptor arm contains distinct information for tRNA-nucleotidyltransferases. However, the absence of an acceptor arm in tRNA^Gly^ (UCC) of *O. nivara* and tRNA^Thr^ (GGU) of *S. bicolor* is quite intriguing. The question arises as to how a tRNA without an acceptor arm can participate to carry an amino acid during the process of protein translation? Some tRNAs contain novel loops having A-C-U-U-U-U-G nucleotides. The stem of the novel loop allows the bonding of A to U and G to U nucleotides. The novel loop structures identified in the present study raises the question whether these loops mimic the anti-codon loop of the tRNA and play a critical role in the protein translation machinery within the chloroplast. Some of the tRNA were also found to contain nine nucleotides in the anti-codon loop; which may represent a novel phenomenon of tRNA. The functional impact of having nine nucleotides in the anti-codon loop remains to be determined. In addition to the presence of few putative novel tRNA structure, chloroplast tRNAs were found to contain a C-A-U anti-codon that codes for tRNA^Ile^ as well. However, the presence of a C-A-U anti-codon in tRNA^Ile^ was previously reported in *Bacillus subtilis* [55].

Phylogenetic analysis of chloroplast tRNA showed two distinct clusters and multiple groupings. Some of the tRNA members of cluster I also found to be present in cluster II; suggesting their evolution by duplication and divergence. However, anti-codon GAC, UAC, GGU, UGU, UGA, GCU, GGA, GCC, UCC, UAA, UAG, CAA, CAU UGC, UCU, and ACG fall independently in the phylogenetic tree; suggesting their evolution from multiple common ancestors. The overlapping grouping of tRNA family members suggests that the tRNAs with these anti-codon groups may have evolved from different common ancestors or may have arisen from duplication events. The presence of tRNA^Met^ twice in cluster I and once in cluster II indicates that tRNA^Met^ is one of the tRNA families that has undergone major duplication event(s) to give rise to other tRNAs. Phylogenetic analysis further revealed that tRNA^Leu^ (CAA), tRNA^Trp^ (CCA), tRNA^Arg^ (UCU), tRNA^Asn^ (GUU), tRNA^Tyr^ (GUA), tRNA^Met^ (CAU), tRNA^Cys^ (GCA), and tRNA^Phe^ (GAA) present in cluster II are the most primitive form of tRNAs with tRNA^Leu^ as the most basal evolutionary ancestor. The grouping of tRNA^Met^ (CAU) with tRNA^Ile^ (CAU), and their similar anti-codon nucleotides, strongly suggests that tRNA^Ile^ evolved directly from tRNA^Met^. The overall analysis clearly indicates that tRNA^Met^ is a major player in the evolution of tRNAs in the chloroplast genome. The distribution of tRNA^Met^ in two different clusters strongly suggests that tRNA^Met^ underwent several major substitution and duplication events to give rise to diverse tRNA families with distinct anti-codons. The rate of transition of chloroplast tRNAs were higher than the rate of transversion. tRNA^Cys^, tRNA^Gln^, tRNA^Trp^, and tRNA^Tyr^ belong to a polar R group and the rate of transversion is zero in tRNAs that carry polar amino acids. Polar amino acids are readily soluble in water and form strong hydrogen bonds with interacting molecules. This suggests that the evolution of chloroplast tRNA^Cys^, tRNA^Gln^, tRNA^Trp^, and tRNA^Tyr^ strongly favors transition substitutions rather than transversion substitutions and that some tRNA Isoacceptors undergo transition more readily than transversion. A few tRNAs, however, underwent a higher rate of transversion than transition; suggesting that the rate of evolution and the rate of transition and transversion of tRNAs are Isoacceptor-specific and that tRNAs have not undergone an equal rate of evolution.

In addition to the mutational event, gene duplication is also a major force in evolution and represents an important mechanism by which species acquire new genes [56]. The majority of novel gene functions have evolved through gene duplication events which can occur by genome duplication, retrotransposons, and unequal crossing over [57, 58]. Ancient duplication events coupled with the retention of extant pairs of duplicated genes have contributed enormously to the evolution of gene families and functional diversification [59]. Plant genomes tend to evolve at a high rate, leading to greater genome diversity relative to other organisms [60]. The study of chloroplast tRNAs showed the rate of deletion of tRNA is superior than the rate of duplication. This suggests that the maternal inheritance of the cyanobacterial-derived chloroplast genome is more intact than the nuclear-encoded plant genome. Therefore, although the species were part of the same lineage, some genes were still lost within each species. This provides further evidence that cyanobacterial tRNAs originated from polyphyletic common ancestors, and hence, loss events are more pronounced than duplication events. Almost all of the tRNAs experienced loss events in either of species studied (Table 5).

**Table 5:** Loss event of chloroplast tRNA in plants

tRNA Gene ID	Species	tRNA	Anti-codon	Lost in Species
NC_002762.1	*T. aestivum*	Val	GAC	*Z. mays*
NC_008602.1	*S. bicolor*	Val	GAC	*Z. mays*, *T. aestivum*
NC_006084.1	*S. officinarum*	Val	GAC	*Z. mays*, *S. bicolor*, *T. aestivum*
NC_001320.1	*O. sativa*	Val	GAC	*O. nivara*
3782	*O. sativa*	Val	GAC	*O. nivara*, *S. bicolor*, *S. officinarum*, *T. aestivum*, *Z. mays*
6258	*O. nivara*	Val	GAC	*O. sativa*, *S. bicolor*, *S. officinarum*, *T. aestivum, Z. mays*
6217	*O. nivara*	Val	GAC	*O. nivara*, *S. bicolor*, *S. officinarum*, *T. aestivum*, *Z. mays*
6160	O. nivara	Val	UAC	*O. sativa*
2026	*Z. mays*	Val	UAC	*S. bicolor*
NC_002762.1	*T. aestivum*		GAC	
6601	*S. officinarum*		UAC	
6160	*O. nivara*		UAC	
2085	*Z. mays*	Ala	UGC	*S. bicolor*
6221	*O. nivara*	Ala	UGC	
5101	*T. aestivum*	Ala	UGC	
5076	*T. aestivum*	Ala	UGC	
6691	*S. officinarum*	Ala	UGC	
6662	*S. officinarum*	Ala	UGC	
3815	*O. sativa*	Ala	UGC	
3786	*O. sativa*	Ala	UGC	
6221	*O. nivara*	Ala	UGC	
2114	*Z. mays*	Ala	UGC	
6253	*O. nivara*	Ala	UGC	
2085	*Z. mays*	Ala	UGC	*O. sativa*
6221	*O. nivara*		UGC	
5076	*T. aestivum*	Ala	UGC	*Z. mays*, *O. nivara*, *O. sativa*
6662	*S. officinarum*	Ala	UGC	*Z. mays*, *O. sativa*, *O. nivara*, *T. aestivum*
3815	*O. sativa*	Ala	UGC	*O. nivara*, *S. officinarum*, *T. aestivum*, *Z. mays*
3786	*O. sativa*	Ala	UGC	*O. nivara*, *S. officinarum*, *T. aestivum*, *Z. mays*
6221	*O. nivara*	Ala	UGC	*O. sativa*, *S. officinarum*, *T. aestivum*, *Z. mays*
2114	*Z. mays*	Ala	UGC	*O. sativa*, *O. nivara*, *S. officinarum*, *T. aestivum*
6253	*O. nivara*	Ala	UGC	*O. sativa*, *S. officinarum*, *T. aestivum*, *Z. mays*
NC_001320.1	*O. sativa*	Thr	GGU	*O. nivara*
6132	*O. nivara*	Thr	GGU	*O. sativa*, *S. officinarum*, *T. aestivum*, *Z. mays*
1998	*Z. mays*	Thr	GGU	*O. nivara*, *O. sativa*, *S. bicolor*, *S. officinarum*, *T. aestivum*
NC_001320.1	*O. sativa*	Thr	UGU	*O. nivara*
6154	*O. nivara*	Thr	UGU	*O. sativa*, *S. bicolor*, *S. officinarum*, *T. aestivum*, *Z. mays*
3729	*O. sativa*	Met	CAU	*O. nivara*
6161	*O. nivara*	Met	CAU	*O. sativa*, *S. bicolor*, *S. officinarum*, *T. aestivum*, *Z. mays*
NC_001320.1	*O. sativa*	Asp	GUC	*O. nivara*
6135	*O. nivara*	Asp	GUC	*O. nivara*, *O. sativa*, *S. bicolor*, *S. officinarum*, *T. aestivum*
6115	*O. nivara*	Lys	UUU	*O. sativa*
5075	*T. aestivum*	Ile	GAU	*Z. mays*
6660	*S. officinarum*	Ile	GAU	*T. aestivum*, *Z. mays*
3816	*O. sativa*	Ile	GAU	*O. nivara*
3784	*O. sativa*	Ile	GAU	*O. nivara*, *S. officinarum*, *T. aestivum*, *Z. mays*
6255	*O. nivara*	Ile	GAU	*O. sativa*
6219	*O. nivara*	Ile	GAU	*O. sativa*, *S. officinarum*, *T. aestivum*, *Z. mays*
6556	*S. officinarum*	Lys	UUU	*S. bicolor*
6115	*O. nivara*	Lys	UUU	
1982	*Z. mays*	Lys	UUU	
4982	*T. aestivum*	Lys	UUU	
2116	*Z. mays*	Ile	GAU	
2083	*Z. mays*	Ile	GAU	
5102	*T. aestivum*	Ile	GAU	
5075	*T. aestivum*	Ile	GAU	
6693	*S. officinarum*	Ile	GAU	
NC_006084.1	*S. officinarum*	Ile	GAU	
3816	*O. sativa*	Ile	GAU	
3784	*O. sativa*	Ile	GAU	
6255	*O. nivara*	Ile	GAU	
6219	*O. nivara*	Ile	GAU	
3723	*O. sativa*	Leu	UAA	*O. nivara*
6155	*O. nivara*	Leu	UAA	*O. sativa*, *S. officinarum*
NC_001320.1	*O. sativa*	Ser	UGA	*O. nivara*
6125	*O. nivara*	Ser	UGA	*O. sativa*, *S. bicolor*, *S. officinarum*, *T. aestivum*, *Z. mays*
3686	*O. sativa*	Ser	UGA	*O. nivara*
6121	*O. nivara*	Ser	GCU	*O. sativa*, *S. bicolor*, *S. officinarum*, *T. aestivum*, *Z. mays*
3720	*O. sativa*	Ser	GGA	*O. nivara*
6152	*O. nivara*	Ser	GGA	*O. sativa*, *S. bicolor*, *S. officinarum*, *T. aestivum*, *Z. mays*
3748	*O. sativa*	Pro	UGG	*O. nivara*
6178	*O. nivara*	Pro	UGG	*O. sativa*, *S. bicolor*, *S. officinarum*, *T. aestivum*, *Z. mays*
3683	*O. sativa*	Gln	UUG	*O. nivara*
6118	*O. nivara*	Gln	UUG	*O. sativa*, *S. bicolor*, *S. officinarum*, *T. aestivum*, *Z. mays*
6178	*O. nivara*	Gln	UGG	*O. sativa*, *S. bicolor*, *S. officinarum*, *T. aestivum*, *Z. mays*
5066	*T. aestivum*	His	GUG	*Z. mays*
NC_008602.1	*S. bicolor*	His	GUG	*Z. mays*, *T. aestivum*
NC_006084.1	*S. officinarum*	His	GUG	*S. bicolor*, *T. aestivum*, *Z. mays*
3833	*O. sativa*	His	GUG	*O. nivara*
3770	*O. sativa*	His	GUG	*O. nivara*, *S. bicolor*, *S. officinarum*, *T. aestivum*, *Z. mays*
6275	*O. nivara*	His	GUG	*O. sativa*, *S. bicolor*, *S. officinarum*, *T. aestivum*, *Z. mays*
6129	*O. nivara*	Gly	UCC	*O. sativa*
3701	*O. sativa*	Glu	UUC	*O. nivara*
6133	*O. nivara*	Glu	UUC	*O. sativa*, *T. aestivum*
3694	*O. sativa*	Met	CAU	*O. nivara*
6128	*O. nivara*	Met	CAU	*O. sativa*, *S. bicolor*, *S. officinarum*, *T. aestivum*, *Z. mays*
5015	*T. aestivum*	Met	CAU	*O. nivara*, *O. sativa*, *S. bicolor*, *S. officinarum*, *Z. mays*
5080	*T. aestivum*	Arg	ACG	*Z. mays*
20,473	*S. bicolor*	Arg	ACG	*T. aestivum*, *Z. mays*
NC_006084.1	*S. officinarum*	Arg	ACG	*S. bicolor*, *T. aestivum*, *Z. mays*
NC_001320.1	*O. sativa*	Arg	ACG	*O. nivara*
3790	*O. sativa*	Arg	ACG	*O. nivara*, *O. sativa*, *S. bicolor*, *S. officinarum*, *Z. mays*
6249	*O. nivara*	Arg	ACG	*O. sativa*, *S. bicolor*, *S. officinarum*, *T. aestivum*, *Z. mays*
3724	*O. sativa*	Phe	GAA	*O. nivara*
6156	*O. nivara*	Phe	GAA	*O. sativa*, *S. bicolor*, *S. officinarum*, *T. aestivum*, *Z. mays*
3706	*O. sativa*	Cys	GCA	*O. nivara*
6138	*O. nivara*	Cys	GCA	*O. sativa*, *T. aestivum*
5069	*T. aestivum*	Met	CAU	*Z. mays*
NC_008602.1	*S. bicolor*	Met	CAU	*T. aestivum*, *S. bicolor*
NC_006084.1	*S. officinarum*	Met	CAU	*S. bicolor*, *T. aestivum*, *Z. mays*
3828	*O. sativa*	Met	CAU	*O. nivara*
3774	*O. sativa*	Met	CAU	*O. nivara*, *S. bicolor*, *S. officinarum*, *T. aestivum*, *Z. mays*
6270	*O. nivara*	Met	CAU	*O. sativa*, *S. bicolor*, *S. officinarum*, *T. aestivum*, *Z. mays*
6206	*O. nivara*	Met	CAU	*O. sativa*, *S. bicolor*, *S. officinarum*, *T. aestivum*, *Z. mays*
6134	*O. nivara*	Tyr	GUA	*O. sativa*
3702	*O. sativa*	Tyr	GUA	*O. nivara*, *S. bicolor*, *S. officinarum*, *T. aestivum*, *Z. mays*
5081	*T. aestivum*	Asn	GUU	*Z. mays*
NC_008602.1	*S. bicolor*	Asn	GUU	*T. aestivum*, *Z. mays*
NC_006084.1	*S. officinarum*	Asn	GUU	*S. bicolor*, *T. aestivum*, *Z. mays*
3809	*O. sativa*	Asn	GUU	*O. nivara*
3792	*O. sativa*	Asn	GUU	*O. nivara*, *S. bicolor*, *S. officinarum*, *T. aestivum*, *Z. mays*
6247	*O. nivara*	Asn	GUU	*O. sativa*, *S. bicolor*, *S. officinarum*, *T. aestivum*, *Z. mays*
6228	*O. nivara*	Asn	GUU	*O. sativa*, *S. bicolor*, *S. officinarum*, *T. aestivum*, *Z. mays*
3715	*O. sativa*	Arg	UCU	*O. nivara*
6147	*O. nivara*	Arg	UCU	*O. sativa*, *S. bicolor*, *S. officinarum*, *T. aestivum*, *Z. mays*
3747	*O. sativa*	Trp	CCA	*O. nivara*
6177	*O. nivara*	Trp	CCA	*O. sativa*, *S. bicolor*, *S. officinarum*, *T. aestivum*, *Z. mays*
5070	*T. aestivum*	Leu	CAA	*Z. mays*
NC_008602.1	*S. bicolor*	Leu	CAA	*T. aestivum*, *Z. mays*
NC_006084.1	*S. officinarum*	Leu	CAA	*S. bicolor*, *T. aestivum*, *Z. mays*
3825	*O. sativa*	Leu	CAA	*O. nivara*
3777	*O. sativa*	Leu	CAA	*O. nivara*, *S. bicolor*, *S. officinarum*, *T. aestivum*, *Z. mays*
6263	*O. nivara*	Leu	CAA	*O. sativa*, *S. bicolor*, *S. officinarum*, *T. aestivum*, *Z. mays*
6212	*O. nivara*	Leu	CAA	*O. sativa*, *S. bicolor*, *S. officinarum*, *T. aestivum*, *Z. mays*
2124	*Z. mays*	Leu	CAA	*O. nivara*, *O. sativa*, *S. bicolor*, *S. officinarum*, *T. aestivum*

## Conclusion

We conducted a tRNA analysis of the chloroplast genome of six monocot plants and found that the chloroplast genome in these plant species encode 28 to 39 tRNA genes. The numbers of tRNA Isoacceptors ranged from 23 to 29 and the majority of tRNAs were associated with only one Isoacceptor. The tRNAs in the chloroplast genome were also found to contain a group I intron in the anti-codon region and a phylogenetic analysis revealed that the chloroplast tRNAs in monocot plants evolved from multiple common ancestors. The chloroplast genomes of the examined monocot plant species were also found to contain putative, novel tRNAs which need to be further investigated to understand their biological significance. An analysis of gene duplication and loss events revealed that gene loss events were more pronounced than duplication events in chloroplast tRNA.

## Methods

### Identification and analysis of chloroplast tRNA of monocot plants

The chloroplast genomes of the monocot species, *O. nivara* (NC_005973), *O. sativa* (NC_001320), *S. officinarum* (NC_006084), *S. bicolor* (NC_008602), *T. aestivum* (NC_002762), and *Z. mays* (NC_001666) were downloaded from the public database available at the National Center for Biotechnology Information (NCBI, https://www.ncbi.nlm.nih.gov/) [46, 61, 62]. The sequences were downloaded in FASTA format (Additional file 1: Table S1, Additional file 3: Data S1) and subsequently all of the chloroplast genomes were subjected to annotation. Annotation of all the chloroplast genomes was carried out using GeSeq-Annotation of Organellar Genomes (https://chlorobox.mpimp-golm.mpg.de/geseq.html) [63]. Parameters used to carry out the annotation process were circular sequence (s); sequence source, chloroplast; generate multi FASTA; annotate plastid IR, BLAT protein search identity 25%; BLAT rRNA, tRNA and DNA search 85% identity; HMMER profile search; Embryophyta chloroplast (CDS + rRNA); 3rd party tRNA annotator ARAGRON v1.2.38, ARWEN v1.2.3, tRNAScan-SE v2.0; and no Refseq selection were utilized. Annotated nucleotide sequences of the chloroplast tRNA genes in the six-monocot species were collected and used in the further sections of this study. The free energy calculation of predicted novel tRNAs were performed using the RNAalifold webserver with default parameters [64].

### Analysis of chloroplast tRNA of monocot plants

The collected genomic tRNA sequences of chloroplast tRNAs of monocot plants were subjected to further analysis using ARAGRON and the tRNAscan-Se server [65]. Default parameters were used to analyze the genomic tRNA sequences in ARAGRON. In the tRNAscan-Se server, the following parameters were used to analyze the genomic tRNA; sequence source, bacterial; search mode, default; query sequences, formatted (FASTA); and genetic code for tRNA isotype prediction, universal. All of the tRNAs were analyzed using the same parameters and the number and composition of nucleotides in different arms and loops were recorded individually. The tRNAs that were found to have a different structure than the canonical clover leaf-like structure characteristic of tRNA were considered as putative novel tRNAs.

### Multiple sequence alignment

To identify and analyze the conserved nucleotide sequences of tRNA isotypes, the nucleotide sequences of 20 isotypes were separately grouped. Later, tRNA isotypes were subjected to multiple sequence alignment using the Multalin server. All of the sequences, in FASTA format, were used in the alignment analysis with the following parameters; sequence input format, auto; display of sequence alignment, colored; alignment matrix, Blosum61–12-2; gap penalty at opening and extension, default; gap penalty at extremities, none and one iteration only, none. The highest alignment consensus value was maintained at 90% (default); whereas, the lowest consensus value was kept at 50% (default). In the displayed alignments, red indicates a similarity/conservation of 90% or more; whereas, blue indicates a sequence conservation less than 90%. Alignments displayed in black indicates no conservation.

### Construction of phylogenetic tree

To analyze the evolution of chloroplast tRNAs in monocot plants, a phylogenetic tree was constructed using MEGA6.0 software [66]. Prior to construction of the phylogenetic tree, a Clustal file of all the tRNAs was created using the Clustal omega server. The generated Clustal file of tRNAs was converted to a MEGA file format using MEGA6 software. Model selection was performed prior to the construction of the phylogenetic tree. Model selection was conducted by MEGA6 software using the following statistical parameters: analysis, model selection (ML); tree to use, automatic (neighbor-joining); statistical method, maximum likelihood; substitution type, nucleotide; gaps/missing data treatment, partial deletion and site coverage cutoff was 95%. The model selection analysis that resulted in the lowest Bayesian information criterion (BIC) was considered as the best model to construct the phylogenetic tree. The lowest BIC score was found to be 7785.682 for the Kimura2+ G + I model; as a result, the latter model was used to construct a phylogenetic tree. Other statistical parameters within the Kimura2+ G + I model were: analysis, phylogeny reconstruction; statistical model, maximum likelihood; test of phylogeny, bootstrap method; no. of bootstrap replicates, 1000; substitution type, nucleotides; rates among sites, Gamma distributed with invariant sites (G + I), no of discrete Gamma categories, 5; gaps/missing data treatment, partial deletion; site coverage cutoff, 95%; and branch swap filter, very strong.

### Analysis of transition and transversion

The MEGA file format of tRNAs used to construct the phylogenetic tree was used to analyze the transition/transversion rate for all of the tRNAs. Additionally, transition/transversion rates of all of the 20 tRNA isotypes were separately studied. The tRNA isotypes were also subjected to multiple sequence alignment using the Clustal omega server to generate a Clustal file for each individual isotype. The generated Clustal files of tRNA isotypes were converted to a MEGA file format and the rate of substitution was estimated using MEGA6 software. The following statistical parameters were used to study the transition/transversion rates in the chloroplast tRNAs of monocot plants: analysis, substitution pattern estimation (ML); tree to use, automatic (neighbor-joining tree); statistical method, maximum likelihood; substitution type, nucleotide; model/method, Kimura2-parameter model; rates among sites, Gamma distributed (G); no. of discrete Gamma categories, 5; gaps/missing data treatment, partial deletion, site coverage cutoff 95%, and branch swap filter, very strong.

### Disparity index analysis

To determine if all of the substitutions of nucleotides occurred homogenously (equal rates) during evolution, a disparity index test of the pattern heterogeneity was conducted to determine the homogeneity of nucleotide substitutions. Statistical parameters used to analyze the pattern of homogeneity were: analysis, disparity index test of substitution pattern homogeneity; scope, in sequence pairs; no. of Monte Carlo Replications, 1000; substitution type, nucleotide; gaps/missing data treatment, partial deletion; and site coverage cutoff was 95%.

### Analysis of gene duplication and loss

An all species tree was first constructed using the NCBI taxonomy browser (https://www.ncbi.nlm.nih.gov/Taxonomy/CommonTree/wwwcmt.cgi) to analyze the duplication and loss events of tRNA genes. Species used to construct the species tree were *O. nivara*, *O. sativa*, *S. officinarum*, *S. bicolor*, *T. aestivum*, and *Z. mays*. The phylogenetic tree used for the evolutionary analysis was utilized as the gene tree. Gene duplication/loss events were studied using Notung2.6 software. The gene tree was reconciled with the species tree during the analysis to obtain the duplication and loss nodes of the genes.

## Additional files

Additional file  1: **Table S1.** Nucleotide composition of acceptor arm, D-arm, D-loop, anti-codon arm, variable loop, pseudouridine arm and pseudouridine loop of chloroplast tRNA. (DOCX 26 kb)

Additional file  2: **Figure S1.** Phylogenetic tree of cyanobacterial tRNAs with tRNAs of *Anabaena cyalindrica*, *Methanococcus maripaludis*, *Methanospirillum hungatei*, *Oscillatoria acuminate*, and *Thermococcus sibiricus*. The tRNAs of these species were included as ingroup, whereas, AtNAC1 and AtNAC2 (NAC transcription factor) of *Arabidopsis thaliana* were used as out-groups. Phylogenetic tree was constructed using the Neighbor-joining method and 1000 bootstrap replicates using MEGA6 software. (PDF 114 kb)

Additional file  3: **Data S1.** tRNA sequences of studied chloroplast genome of the monocot plants. (TXT 24 kb)

## Abbreviations

A: Adenine
C: Cytosine
G: Guanine
tRNA: Transfer RNA
U: Uracil
Ψ: Pseudouridine

## References

[1] Wise RR, Hoober J (2006). The structure and function of plastids. Divers. Plast. Form Funct.

[2] Wolfgang K, Martin H (2001). Uncertainties in global terrestrial biosphere modeling: a comprehensive sensitivity analysis with a new photosynthesis and energy balance scheme. Global Biogeochem Cycles Wiley-Blackwell.

[3] Des Marais DJ (2000). When did photosynthesis emerge on earth?. Science.

[4] Stern DB, Goldschmidt-Clermont M, Hanson MR (2010). Chloroplast RNA metabolism. Annu Rev Plant Biol Annual Reviews.

[5] Bolton JR, Hall DO (1979). Photochemical conversion and storage of solar energy. Annu Rev Energy Annual Reviews.

[6] Jensen WA, Jensen WA (1973). Chloroplasts and photosynthesis. Plant Cell.

[7] Spetea C, Hundal T, Lundin B, Heddad M, Adamska I, Andersson B (2004). Multiple evidence for nucleotide metabolism in the chloroplast thylakoid lumen. Proc Natl Acad Sci U S A . National Academy of Sciences.

[8] Stitt M, Lilley RM, Heldt HW (1982). Adenine nucleotide levels in the cytosol, chloroplasts, and mitochondria of wheat leaf protoplasts. Plant Physiol.

[9] Noctor G, Arisi A-CM, Jouanin L, Foyer CH (1998). Manipulation of glutathione and amino acid biosynthesis in the chloroplast. Plant Physiol.

[10] Schulze-Siebert D, Heineke D, Scharf H, Schultz G (1984). Pyruvate-derived amino acids in spinach chloroplasts. Plant Physiol.

[11] Blee E, Joyard J (1996). Envelope membranes from spinach chloroplasts are a site of metabolism of fatty acid Hydroperoxides. Plant Physiol.

[12] Vick BA, Zimmerman DC (1987). Pathways of fatty acid Hydroperoxide metabolism in spinach leaf chloroplasts. Plant Physiol.

[13] Wallsgrove RM, Keys A, Lea PJ, Miflin BJ (1983). Photosynthesis, photorespiration and nitrogen metabolism. Plant Cell Environ.

[14] Pilon-Smits EAH, Garifullina GF, Abdel-Ghany S, Kato S-I, Mihara H, Hale KL (2002). Characterization of a NifS-like chloroplast protein from Arabidopsis. Implications for its role in sulfur and selenium metabolism. Plant Physiol.

[15] Asahi T (1964). Sulfur metabolism in higher plants: IV. Mechanism of sulfate reduction in chloroplasts. Biochim Biophys Acta.

[16] Martin W, Rujan T, Richly E, Hansen A, Cornelsen S, Lins T (2002). Evolutionary analysis of Arabidopsis, cyanobacterial, and chloroplast genomes reveals plastid phylogeny and thousands of cyanobacterial genes in the nucleus. Proc Natl Acad Sci U S A National Academy of Sciences.

[17] Gray MW (1989). The evolutionary origins of organelles. Trends Genet.

[18] Martin W, Stoebe B, Goremykin V, Hansmann S, Hasegawa M (1998). Kowallik K V. gene transfer to the nucleus and the evolution of chloroplasts. Nature. Macmillan Magazines Ltd.

[19] Falcón LI, Magallón S, Castillo A (2010). Dating the cyanobacterial ancestor of the chloroplast. Isme J. International Society for Microbial Ecology.

[20] Raven JA, Allen JF (2003). Genomics and chloroplast evolution: what did cyanobacteria do for plants? Genome biol. London: BioMed Central.

[21] Kolodner R, Tewari KK (1979). Inverted repeats in chloroplast DNA from higher plants. Proc Natl Acad Sci.

[22] Shinozaki K, Ohme M, Tanaka M, Wakasugi T, Hayashida N, Matsubayashi T. The complete nucleotide sequence of the tobacco chloroplast genome: its gene organization and expression. EMBO J. 1986;5. 10.1002/j.1460-2075.1986.tb04464.x PMC1167080 16453699

[23] Maier RM, Neckermann K, Igloi GL, Kossel H. Complete sequence of the maize chloroplast genome: gene content, hotspots of divergence and fine tuning of genetic information by transcript editing. J Mol Biol. 1995;251. 10.1006/jmbi.1995.0460 7666415

[24] Wang R-J, Cheng C-L, Chang C-C, Wu C-L, Su T-M, Chaw S-M (2008). Dynamics and evolution of the inverted repeat-large single copy junctions in the chloroplast genomes of monocots. BMC Evol Biol.

[25] Zurawski G, Bottomley W, Whitfeld PR (1984). Junctions of the large single copy region and the inverted repeats in Spinacia oleracea and Nicotiana debneyi chloroplast DNA: sequence of the genes for tRNA his and the ribosomal proteins S19 and L2. Nucleic Acids Res.

[26] Hereward JP, Werth JA, Thornby DF, Keenan M, Chauhan BS, Walter GH. Complete chloroplast genome of glyphosate resistant Sonchus oleraceus L. from Australia, with notes on the small single copy (SSC) region orientation. Mitochondrial DNA Part B. 2018;3:363–4. 10.1080/23802359.2018.1450682 PMC7799887 33474170

[27] Kuo L-Y, Tang T-Y, Li F-W, Su H-J, Chiou W-L, Huang Y-M (2018). Organelle genome inheritance in Deparia ferns (Athyriaceae, Aspleniineae, Polypodiales). Front Plant Sci.

[28] Neale DB, Wheeler NC, Allard RW (1986). Paternal inheritance of chloroplast DNA in Douglas-fir. Can J For Res NRC Research Press.

[29] Wolfe KH, Li WH, Sharp PM (1987). Rates of nucleotide substitution vary greatly among plant mitochondrial, chloroplast, and nuclear DNAs. Proc Natl Acad Sci.

[30] Provan J, Soranzo N, Wilson NJ, Goldstein DB, Powell WA (1999). Low mutation rate for chloroplast microsatellites. Genetics.

[31] Leitch IJ, Beaulieu JM, Chase MW, Leitch AR, Fay MF (2010). Genome size dynamics and evolution in monocots. J Bot.

[32] Soltis DE, Bell CD, Kim S, Soltis PS (2008). Origin and early evolution of angiosperms. Ann N Y Acad Sci John Wiley & Sons, Ltd.

[33] Meeuse ADJ. Aspects of the early evolution of the monocotyledons. Acta bot Neerl . John Wiley & Sons, Ltd; 2018;24:421–436.

[34] Holley RW, Apgar J, Everett GA, Madison JT, Marquisee M, Merrill SH (1965). Structure of a ribonucleic acid. Science American Association for the Advancement of Science.

[35] Mohanta TK, Bae H. Analyses of genomic trna reveal presence of novel tRNAs in oryza sativa. Front Genet. 2017;8. 10.3389/fgene.2017.00090 PMC5492330 28713421

[36] Goodenbour JM, Pan T (2006). Diversity of tRNA genes in eukaryotes. Nucleic Acids Res.

[37] Kirchner S, Ignatova Z (2014). Emerging roles of tRNA in adaptive translation, signalling dynamics and disease. Nat Rev Genet Nature Publishing Group.

[38] Maréchal-Drouard L, Guillemaut P, Pfitzingzer H, Weil JH, Mache R, Stutz E, Subramanian AR (1991). Chloroplast tRNAs and tRNA genes: structure and function. Transl Appar Photosynth organelles.

[39] Laslett D, Canbäck BARAGORN (2004). A program to detect tRNA genes and tmRNA genes in nucleotide sequences. Nucleic Acids Res.

[40] Dieci G, Fiorino F, Castelnuovo M, Teichmann M, Pagano A. The expanding RNA polymerase III transcriptome. Trends Genet. 23(12):614–22. 10.1016/j.tig.2007.09.001 17977614

[41] Lyons E, Pedersen B, Kane J, Alam M, Ming R, Tang H (2008). Finding and comparing Syntenic regions among Arabidopsis and the outgroups papaya, poplar, and grape: CoGe with Rosids. Plant Physiol.

[42] SD E, AV A, Jim L, BC D, PA H, Chunfang Z (2009). Polyploidy and angiosperm diversification. Am J Bot Wiley-Blackwell.

[43] Lee T-H, Tang H, Wang X, Paterson AH. PGDD: A database of gene and genome duplication in plants. Nucleic Acids Res . Oxford University Press; 2013;41:D1152–D1158. 10.1093/nar/gks1104 PMC3531184 23180799

[44] Simon R, WJ F (2014). Doubling down on genomes: polyploidy and crop plants. Am J Bot Wiley-Blackwell.

[45] Mohanta TK, Syed AS, Ameen F, Bae H. Novel genomic and evolutionary perspective of cyanobacterial tRNAs. Front Genet. 2017;8. 10.3389/fgene.2017.00200 PMC5733544 29321793

[46] Hiratsuka J, Shimada H, Whittier R, Ishibashi T, Sakamoto M, Mori M. The complete sequence of the rice (Oryza sativa) chloroplast genome: intermolecular recombination between distinct tRNA genes accounts for a major plastid DNA inversion during the evolution of the cereals. Mol Gen Genet. 1989;217. 10.1007/BF02464880 2770692

[47] Howe CJ (1985). The endpoints of an inversion in wheat chloroplast DNA are associated with short repeated sequences containing homology toatt-lambda. Curr Genet.

[48] Kozak M (1999). Initiation of translation in prokaryotes and eukaryotes. Gene.

[49] Guillon JM, Mechulam Y, Schmitter JM, Blanquet S, Fayat G (1992). Disruption of the gene for met-tRNA(f)/(met) formyltransferase severely impairs growth of Escherichia coli. J Bacteriol.

[50] Varshney U, Lee CP, Seong BL, RajBhandary UL (1991). Mutants of initiator tRNA that function both as initiators and elongators. J Biol Chem.

[51] Salinas-Giegé T, Giegé R, Giegé P (2015). tRNA biology in mitochondria. Ibba M, editor Int J Mol Sci MDPI.

[52] Sharp SJ, Schaack J, Cooley L, Burke DJ, Soil D (1985). Structure and transcription of eukaryotic tRNA gene. Crit Rev Biochem Taylor & Francis.

[53] Paquin B, Kathe SD, Shub DA, Paquin B, Kathe SD, Nierzwicki-bauer SA (1997). Origin and evolution of group I introns in cyanobacterial tRNA genes . Origin and evolution of group I introns in cyanobacterial tRNA genes. J Bacteriol.

[54] Rudi K, Jacobsen KS. Cyanobacterial tRNA(Leu)(UAA) group I introns have polyphyletic origin. FEMS Microbiol Lett. 1997;156(2):293-8. 10.1016/s0378-1097(97)00446-1 9513279

[55] Köhrer C, Mandal D, Gaston KW, Grosjean H, Limbach PA, RajBhandary UL. Life without tRNA(Ile)-lysidine synthetase: translation of the isoleucine codon AUA in Bacillus subtilis lacking the canonical tRNA(2)(Ile). Nucleic Acids Res. 2014;42:1904–915. 10.1093/nar/gkt1009 PMC3919564 24194599

[56] Magadum S, Banerjee U, Murugan P, Gangapur D, Ravikesavan R (2013). Gene duplication as a major force in evolution. J Genet.

[57] Silver L, Genet E (2001). Evolution of gene families. Miller JHBT-E of G.

[58] Carroll D, Duplication G, Genet E (2001). Miller JHBT-E of G.

[59] Panchy N, Lehti-Shiu M, Shiu S-H (2016). Evolution of gene Duplication in plants. Plant Physiol American Society of Plant Biologists.

[60] Kejnovsky E, Leitch IJ, Leitch AR (2009). Contrasting evolutionary dynamics between angiosperm and mammalian genomes. Trends Ecol Evol Elsevier.

[61] Shahid Masood M, Nishikawa T, Fukuoka S, Njenga PK, Tsudzuki T, Kadowaki K (2004). The complete nucleotide sequence of wild rice (Oryza nivara) chloroplast genome: first genome wide comparative sequence analysis of wild and cultivated rice. Gene.

[62] Saski C, Lee SB, Fjellheim S, Guda C, Jansen RK, Luo H. Complete chloroplast genome sequences of Hordeum vulgare, Sorghum bicolor and Agrostis stolonifera, and comparative analyses with other grass genomes. Theor Appl Genet. 2007;115. 10.1007/s00122-007-0567-4 PMC2674615 17534593

[63] Tillich M, Lehwark P, Pellizzer T, Ulbricht-Jones ES, Fischer A, Bock R (2017). GeSeq – versatile and accurate annotation of organelle genomes. Nucleic Acids Res.

[64] Bernhart SH, Hofacker IL, Will S, Gruber AR, Stadler PF (2008). RNAalifold: improved consensus structure prediction for RNA alignments. BMC Bioinformatics.

[65] Lowe TM, Chan PP. tRNAscan-SE on-line: integrating search and context for analysis of transfer RNA genes. Nucleic Acids Res . Oxford University Press; 2016;44:W54–W57. 10.1093/nar/gkw413 PMC4987944 27174935

[66] Tamura K, Stecher G, Peterson D, Filipski A, Kumar S (2013). MEGA6: molecular evolutionary Genetics analysis version 6.0. Mol Biol Evol.

