# Supplementary material for: Genomic and evolutionary aspects of chloroplast tRNA in monocot plants
Source: BMC Plant Biol. 2019 Jan 22;19:39. doi: 10.1186/s12870-018-1625-6 (PMC6341768; doi:10.1186/s12870-018-1625-6)
Supplement: Supplementary file 1 — Table S1. Nucleotide composition of acceptor arm, D-arm, D-loop, anti-codon arm, variable loop, pseudouridine arm and pseudouridine loop of chloroplast tRNA. (DOCX 26 kb) [file 12870_2018_1625_MOESM1_ESM.docx]

**Additional file 3: Table S1**

Nucleotide composition of acceptor arm, D-arm, D-loop, anti-codon arm, variable loop, pseudouridine arm and pseudouridine loop of chloroplast tRNA.

|  | AA | DA | DL | ACA | ACL | VL | ΨA | ΨL |
| --- | --- | --- | --- | --- | --- | --- | --- | --- |
| Alanine | | | | | | | | |
| Oryza_nivara_6253_OrniCt031 | 7 | 4 | 8 | 5 | 7 | 5 | 5 | 7 |
| Oryza_nivara_6221_OrniCt025 | 7 | 4 | 8 | 5 | 7 | 5 | 5 | 7 |
| Oryza_sativa_3786_OrsajCt150 | 7 | 4 | 8 | 5 | 7 | 5 | 5 | 7 |
| Oryza_sativa_3815_OrsajCt156 | 7 | 4 | 8 | 5 | 7 | 5 | 5 | 7 |
| Saccharum_officinarum_6662 | 7 | 4 | 8 | 5 | 7 | 5 | 5 | 7 |
| Saccharum_officinarum_6691 | 7 | 4 | 8 | 5 | 7 | 5 | 5 | 7 |
| Triticum_aestivum_5076 | 7 | 4 | 8 | 5 | 7 | 5 | 5 | 7 |
| Triticum_aestivum_5101 | 7 | 4 | 8 | 5 | 7 | 5 | 5 | 7 |
| Zea_mays_2085 | 7 | 4 | 8 | 5 | 7 | 5 | 5 | 7 |
| Zea_mays_2114 | 7 | 4 | 8 | 5 | 7 | 5 | 5 | 7 |
| Arginine | | | | | | | | |
| Oryza_nivara_6147 | 7 | 4 | 8 | 5 | 7 | 4 | 5 | 7 |
| Oryza_nivara_6221 | 7 | 4 | 8 | 5 | 7 | 5 | 5 | 7 |
| Oryza_nivara_6249 | 7 | 4 | 9 | 5 | 7 | 5 | 5 | 7 |
| Oryza_sativa_3715 | 7 | 4 | 8 | 5 | 7 | 4 | 5 | 7 |
| Oryza_sativa_3790 | 7 | 4 | 9 | 5 | 7 | 5 | 5 | 7 |
| Oryza_sativa_3811 | 7 | 4 | 9 | 5 | 7 | 5 | 5 | 7 |
| Saccharum_officinarum_6588 | 7 | 4 | 8 | 5 | 7 | 4 | 5 | 7 |
| Saccharum_officinarum_6667 | 7 | 4 | 9 | 5 | 7 | 5 | 5 | 7 |
| Saccharum_officinarum_6687 | 7 | 4 | 9 | 5 | 7 | 5 | 5 | 7 |
| Sorghum_bicolor_20401 | 7 | 4 | 8 | 5 | 7 | 4 | 5 | 7 |
| Sorghum_bicolor_20473 | 7 | 4 | 9 | 5 | 7 | 5 | 5 G | 7 |
| Sorghum_bicolor_20489 | 7 | 4 | 9 | 5 | 7 | 5 | 5 | 7 |
| Triticum_aestivum_5014 | 7 | 4 | 8 | 5 | 7 | 4 | 5 | 7 |
| Triticum_aestivum_5080 | 7 | 4 | 9 | 5 | 7 | 5 | 5 G | 7 |
| Triticum_aestivum_5097 | 7 | 4 | 9 | 5 | 7 | 5 | 5 G | 7 |
| Zea_mays_2013 | 7 | 4 | 8 | 5 | 7 | 5 | 5 | 7 |
| Zea_mays_2090 | 7 | 4 | 9 | 5 | 7 | 5 | 5G | 7 |
| Asparagine | | | | | | | | |
| Oryza_nivara_6228 | 7 | 4 | 7 | 5 | 7 | 5 | 5 | 7 |
| Oryza_nivara_6247 | 7 | 4 | 7 | 5 | 7 | 5 | 5 | 7 |
| Oryza_sativa_3792 | 7 | 4 | 7 | 5 | 7 | 5 | 5 | 7 |
| Oryza_sativa_3809 | 7 | 4 | 7 | 5 | 7 | 5 | 5 | 7 |
| Saccharum_officinarum_6668 | 7 | 4 | 7 | 5 | 7 | 5 | 5 | 7 |
| Saccharum_officinarum_6686 | 7 | 4 | 7 | 5 | 7 | 5 | 5 | 7 |
| Sorghum_bicolor_20474 | 7 | 4 | 7 | 5 | 7 | 5 | 5 | 7 |
| Sorghum_bicolor_20488 | 7 | 4 | 7 | 5 | 7 | 5 | 5 | 7 |
| Triticum_aestivum_5081 | 7 | 4 | 7 | 5 | 7 | 5 | 5 | 7 |
| Triticum_aestivum_5096 | 7 | 4 | 7 | 5 | 7 | 5 | 5 | 7 |
| Zea_mays_2092 | 7 | 4 | 7 | 5 | 7 | 5 | 5 | 7 |
| Zea_mays_2108 | 7 | 4 | 7 | 5 | 7 | 5 | 5 | 7 |
| Aspartate | | | | | | | | |
| Oryza_nivara_6135 | 7 | 4 | 9 | 5 | 7 | 5 | 5 | 7 |
| Oryza_sativa_3703 | 7 | 4 | 9 | 5 | 7 | 5 | 5 | 7 |
| Saccharum_officinarum_6576 | 7 | 4 | 9 | 5 | 7 | 5 | 5 | 7 |
| Sorghum_bicolor_20389 | 7 | 4 | 9 | 5 | 7 | 5 | 5 | 7 |
| Triticum_aestivum_5002 | 7 | 4 | 9 | 5 | 7 | 5 | 5 | 7 |
| Zea_mays_2001 | 7 | 4 | 9 | 5 | 7 | 5 | 5 | 7 |
| Cysteine | | | | | | | | |
| Oryza_nivara_6138 | 7 | 3 | 9 | 5 | 7 | 4 | 5 | 7 |
| Oryza_sativa_3706 | 7 | 3 | 9 | 5 | 7 | 4 | 5 | 7 |
| Saccharum_officinarum_6579 | 7 | 3 | 9 | 5 | 7 | 4 | 5 | 7 |
| Sorghum_bicolor_20392 | 7 | 3 | 9 | 5 | 7 | 4 | 5 | 7 |
| Triticum_aestivum_5005 | 7 | 3 | 9 | 5 | 7 | 4 | 5 | 7 |
| Zea_mays_2004 | 7 | 3 | 9 | 5 | 7 | 4 | 5 | 7 |
| Glutamate | | | | | | | | |
| Oryza_nivara_6133 | 7 | 4 | 9 | 5 | 7 | 4 | 5 | 7 |
| Oryza_sativa_3701 | 7 | 4 | 9 | 5 | 7 | 4 | 5 | 7 |
| Saccharum_officinarum_6574 | 7 | 4 | 9 | 5 | 7 | 4 | 5 | 7 |
| Sorghum_bicolor_20387 | 7 | 4 | 9 | 5 | 7 | 4 | 5 | 7 |
| Triticum_aestivum_5000 | 7 | 4 | 9 | 5 | 7 | 4 | 5 | 7 |
| Zea_mays_1999 | 7 | 4 | 9 | 5 | 7 | 4 | 5 | 7 |
| Glutamine | | | | | | | | |
| Oryza_nivara_6118 | 7 | 3 | 9 | 5 | 7 | 5 | 5 | 7 |
| Oryza_sativa_3683 | 7 | 3 | 9 | 5 | 7 | 5 | 5 | 7 |
| Saccharum_officinarum_6559 | 7 | 3 | 9 | 5 | 7 | 5 | 5 | 7 |
| Sorghum_bicolor_20373 | 7 | 3 | 9 | 5 | 7 | 5 | 5 | 7 |
| Triticum_aestivum_4985 | 7 | 3 | 9 | 5 | 7 | 5 | 5 | 7 |
| Zea_mays_1985 | 7 | 3 | 9 | 5 | 7 | 5 | 5 | 7 |
| Glycine | | | | | | | | |
| Oryza_nivara_6127 | 7 | 3 | 9 | 5 | 7 | 5 | 4 | 7 |
| Oryza_nivara_6129 | 1 | 4 | 8 | 4 | 7 | 6 | 5 | 7 |
| Oryza_sativa_3692 | 7 | 3 | 9 | 5 | 7 | 5 | 4 | 7 |
| Saccharum_officinarum_6568 | 7 | 3 | 9 | 5 | 7 | 4 | 5 | 7 |
| Saccharum_officinarum_6570 | 7 | 4 | 7 | 4 | 7 | 5 | 5 | 7 |
| Sorghum_bicolor_20381 | 7 | 3 | 9 | 5 | 7 | 4 | 5 | 7 |
| Triticum_aestivum_4993 | 7 | 3 | 9 | 5 | 7 | 5 | 4 | 7 |
| Triticum_aestivum_4995 | 7 | 4 | 8 | 4 | 7 | 5 | 5 | 7 |
| Zea_mays_1993 | 7 | 3 | 9 | 5 | 7 | 4 | 5 | 7 |
| Zea_mays_1995 | 7 | 4 | 7 | 4 | 7 | 5 | 5 | 7 |
| Histidine | | | | | | | | |
| Oryza_nivara_6275 | 7 | 3 | 11 | 5 | 7 | 5 | 5 | 7 |
| Oryza_sativa_3770 | 7 | 3 | 11 | 5 | 7 | 5 | 5 | 7 |
| Oryza_sativa_3833 | 7 | 3 | 11 | 5 | 7 | 5 | 5 | 7 |
| Saccharum_officinarum_6640 | 7 | 3 | 11 | 5 | 7 | 5 | 5 | 7 |
| Saccharum_officinarum_6715 | 7 | 3 | 11 | 5 | 7 | 5 | 5 | 7 |
| Sorghum_bicolor_20366 | 7 | 3 | 11 | 5 | 7 | 5 | 5 | 7 |
| Sorghum_bicolor_20457 | 7 | 3 | 11 | 5 | 7 | 5 | 5 | 7 |
| Triticum_aestivum_5066 | 7 | 3 | 11 | 5 | 7 | 5 | 5 | 7 |
| Triticum_aestivum_5111 | 7 | 3 | 11 | 5 | 7 | 5 | 5 | 7 |
| Zea_mays_2065 | 7 | 3 | 11 | 5 | 7 | 5 | 5 | 7 |
| Zea_mays_2136 | 7 | 3 | 11 | 5 | 7 | 5 | 5 | 7 |
| Ileucine | | | | | | | | |
| Oryza_nivara_6219 | 7 | 4 | 7 | 5 | 7 | 5 | 5 | 7 |
| Oryza_nivara_6255 | 7 | 4 | 7 | 5 | 7 | 5 | 5 | 7 |
| Oryza_sativa_3784 | 7 | 4 | 7 | 5 | 7 | 5 | 5 | 7 |
| Oryza_sativa_3816 | 7 | 4 | 7 | 5 | 7 | 5 | 5 | 7 |
| Saccharum_officinarum_6660 | 7 | 4 | 7 | 5 | 7 | 5 | 5 | 7 |
| Saccharum_officinarum_6693 | 7 | 4 | 7 | 5 | 7 | 5 | 5 | 7 |
| Triticum_aestivum_5075 | 7 | 4 | 7 | 5 | 7 | 5 | 5 | 7 |
| Triticum_aestivum_5102 | 7 | 4 | 7 | 5 | 7 | 5 | 5 | 7 |
| Zea_mays_2069 | 7 | 3 | 7 | 4 | 7 | 7 | 5 | 7 |
| Zea_mays_2083 | 7 | 4 | 7 | 5 | 7 | 5 | 5 | 7 |
| Leucine | | | | | | | | |
| Oryza_nivara_6155 | 7 | 3 | 11 | 5 | 7 | 16 | 5 | 7 |
| Oryza_nivara_6212 | 7 | 3 | 10 | 5 | 7 | 13 | 5 | 7 |
| Oryza_nivara_6235 | 7 | 3 | 11 | 5 | 7 | 11 | 5 | 7 |
| Oryza_nivara_6263 | 7 | 3 | 10 | 5 | 7 | 13 | 5 | 7 |
| Oryza_sativa_3723 | 7 | 3 | 11 | 5 | 7 | 16 | 5 | 7 |
| Oryza_sativa_3777 | 7 | 3 | 10 | 5 | 7 | 13 | 5 | 7 |
| Oryza_sativa_3798 | 7 | 3 | 11 | 5 | 7 | 11 | 5 | 7 |
| Oryza_sativa_3825 | 7 | 3 | 10 | 5 | 7 | 13 | 5 | 7 |
| Saccharum_officinarum_6596 | 7 | 3 | 11 | 5 | 7 | 16 | 5 | 7 |
| Saccharum_officinarum_6652 | 7 | 3 | 10 | 5 | 7 | 13 | 5 | 7 |
| Saccharum_officinarum_6674 | 7 | 3 | 11 | 5 | 7 | 11 | 5 | 7 |
| Saccharum_officinarum_6702 | 7 | 3 | 10 | 5 | 7 | 13 | 5 | 7 |
| Sorghum_bicolor_20461 | 7 | 3 | 10 | 5 | 7 | 13 | 5 | 7 |
| Sorghum_bicolor_20478 | 7 | 3 | 11 | 5 | 7 | 11 | 5 | 7 |
| Sorghum_bicolor_20501 | 7 | 3 | 10 | 5 | 7 | 13 | 5 | 7 |
| Triticum_aestivum_5023 | 7 | 3 | 11 | 5 | 7 | 16 | 5 | 7 |
| Triticum_aestivum_5070 | 7 | 3 | 10 | 5 | 7 | 13 | 5 | 7 |
| Triticum_aestivum_5086 | 7 | 3 | 11 | 5 | 7 | 11 | 5 | 7 |
| Triticum_aestivum_5107 | 7 | 3 | 10 | 5 | 7 | 13 | 5 | 7 |
| Zea_mays_2021 | 7 | 3 | 11 | 5 | 7 | 16 | 5 | 7 |
| Zea_mays_2077 | 7 | 3 | 10 | 5 | 7 | 13 | 5 | 7 |
| Zea_mays_2097 | 7 | 3 | 11 | 5 | 7 | 12 | 5 | 7 |
| Zea_mays_2124 | 7 | 3 | 10 | 5 | 7 | 13 | 5 | 7 |
| Lysine | | | | | | | | |
| Oryza_nivara_6115 | 7 | 4 | 7 | 4 | 7 | 6 | 5 | 7 |
| Saccharum_officinarum_6556 | 7 | 4 | 7 | 4 | 7 | 6 | 5 | 7 |
| Triticum_aestivum_4982 | 7 | 4 | 7 | 4 | 7 | 5 | 5 | 7 |
| Zea_mays_1982 | 7 | 4 | 7 | 5 | 7 | 5 | 5 | 7 |
| Methionine | | | | | | | | |
| Oryza_nivara_6128 | 7 | 4 | 9 | 5 | 7 | 5 | 5 | 7 |
| Oryza_nivara_6161 | 7 | 4 | 8 | 5 | 7 | 5 | 5 | 7 |
| Oryza_nivara_6206 | 7 | 3 | 10 | 4 | 7 | 7 | 5 | 7 |
| Oryza_nivara_6270 | 7 | 3 | 10 | 4 | 7 | 7 | 5 | 7 |
| Oryza_sativa_3694 | 7 | 4 | 9 | 5 | 7 | 5 | 5 | 7 |
| Oryza_sativa_3729 | 7 | 4 | 8 | 5 | 7 | 5 | 5 | 7 |
| Oryza_sativa_3774 | 7 | 3 | 10 | 4 | 7 | 7 | 5 | 7 |
| Oryza_sativa_3828 | 7 | 3 | 10 | 4 | 7 | 7 | 5 | 7 |
| Saccharum_officinarum_6569 | 7 | 4 | 9 | 5 | 7 | 5 | 5 | 7 |
| Saccharum_officinarum_6602 | 7 | 4 | 8 | 5 | 7 | 5 | 5 | 7 |
| Saccharum_officinarum_6644 | 7 | 3 | 10 | 4 | 7 | 7 | 5 | 7 |
| Saccharum_officinarum_6710 | 7 | 3 | 10 | 4 | 7 | 7 | 5 | 7 |
| Sorghum_bicolor_20382 | 7 | 4 | 9 | 5 | 7 | 5 | 5 | 7 |
| Sorghum_bicolor_20418 | 7 | 4 | 8 | 5 | 7 | 5 | 5 | 7 |
| Sorghum_bicolor_20460 | 7 | 3 | 10 | 4 | 7 | 7 | 5 | 7 |
| Sorghum_bicolor_20502 | 7 | 3 | 10 | 4 | 7 | 7 | 5 | 7 |
| Triticum_aestivum_4994 | 6 | 4 | 9 | 5 | 7 | 5 | 5 | 7 |
| Triticum_aestivum_5015 | 5 | 4 | 9 | 5 | 7 | 5 | 5 | 7 |
| Triticum_aestivum_5029 | 7 | 4 | 8 | 5 | 7 | 5 | 5 | 7 |
| Triticum_aestivum_5069 | 7 | 3 | 10 | 4 | 7 | 7 | 5 | 7 |
| Triticum_aestivum_5108 | 7 | 3 | 10 | 4 | 7 | 7 | 5 | 7 |
| Zea_mays_1994 | 7 | 4 | 9 | 5 | 7 | 5 | 5 | 7 |
| Zea_mays_2027 | 7 | 4 | 8 | 5 | 7 | 5 | 5 | 7 |
| Phenylalanine | | | | | | | | |
| Oryza_nivara_6156 | 7 | 4 | 8 | 5 | 7 | 5 | 5 | 7 |
| Oryza_sativa_3724 | 7 | 4 | 8 | 5 | 7 | 5 | 5 | 7 |
| Saccharum_officinarum_6597 | 7 | 4 | 8 | 5 | 7 | 5 | 5 | 7 |
| Sorghum_bicolor_20412 | 7 | 4 | 8 | 5 | 7 | 5 | 5 | 7 |
| Triticum_aestivum_5024 | 7 | 4 | 8 | 5 | 7 | 5 | 5 | 7 |
| Zea_mays_2022 | 7 | 4 | 8 | 5 | 7 | 5 | 5 | 7 |
| Proline | | | | | | | | |
| Oryza_nivara_6178 | 7 | 4 | 9 | 5 | 7 | 5 | 5 | 7 |
| Oryza_sativa_3748 | 7 | 4 | 9 | 5 | 7 | 5 | 5 | 7 |
| Saccharum_officinarum_6617 | 7 | 4 | 9 | 5 | 7 | 5 | 5 | 7 |
| Sorghum_bicolor_20434 | 7 | 4 | 9 | 5 | 7 | 5 | 5 | 7 |
| Triticum_aestivum_5044 | 7 | 4 | 9 | 5 | 7 | 5 | 5 | 7 |
| Zea_mays_2042 | 7 | 4 | 9 | 5 | 7 | 5 | 5 | 7 |
| Serine | | | | | | | | |
| Oryza_nivara_6121 | 7 | 3 | 11 | 5 | 7 | 19 | 5 | 7 |
| Oryza_nivara_6125 | 7 | 4 | 9 | 5 | 7 | 19 | 5 | 7 |
| Oryza_nivara_6152 | 7 | 3 | 11 | 5 | 7 | 18 | 5 | 7 |
| Oryza_sativa_3686 | 7 | 3 | 11 | 5 | 7 | 19 | 5 | 7 |
| Oryza_sativa_3690 | 7 | 4 | 9 | 5 | 7 | 19 | 5 | 7 |
| Oryza_sativa_3720 | 7 | 3 | 11 | 5 | 7 | 18 | 5 | 7 |
| Saccharum_officinarum_6562 | 7 | 3 | 11 | 5 | 7 | 19 | 5 | 7 |
| Saccharum_officinarum_6566 | 7 | 4 | 9 | 5 | 7 | 19 | 5 | 7 |
| Saccharum_officinarum_6593 | 7 | 3 | 11 | 5 | 7 | 18 | 5 | 7 |
| Sorghum_bicolor_20376 | 7 | 4 | 11 | 5 | 7 | 19 | 5 | 7 |
| Sorghum_bicolor_20379 | 7 | 4 | 9 | 5 | 7 | 19 | 5 | 7 |
| Sorghum_bicolor_20407 | 7 | 3 | 11 | 5 | 7 | 18 | 5 | 7 |
| Triticum_aestivum_5020 | 7 | 3 | 11 | 5 | 7 | 18 | 5 | 7 |
| Triticum_aestivum_4988 | 7 | 3 | 11 | 5 | 7 | 19 | 5 | 7 |
| Triticum_aestivum_4991 | 7 | 4 | 9 | 5 | 7 | 19 | 5 | 7 |
| Zea_mays _1988 | 7 | 3 | 11 | 5 | 7 | 19 | 5 | 7 |
| Zea_mays_1991 | 7 | 4 | 9 | 5 | 7 | 19 | 5 | 7 |
| Threonine | | | | | | | | |
| Oryza_nivara_6132 | 7 | 4 | 7 | 5 | 7 | 5 | 5 | 7 |
| Oryza_nivara_6154 | 7 | 4 | 8 | 5 | 7 | 5 | 5 | 7 |
| Oryza_sativa_3699 | 7 | 4 | 7 | 5 | 7 | 5 | 5 | 7 |
| Oryza_sativa_3722 | 7 | 4 | 8 | 5 | 7 | 5 | 5 | 7 |
| Saccharum_officinarum_6573 | 7 | 4 | 7 | 5 | 7 | 5 | 5 | 7 |
| Saccharum_officinarum_6595 | 7 | 4 | 8 | 5 | 7 | 5 | 5 | 7 |
| Sorghum_bicolor_20385 | 1 | 4 | 7 | 5 | 7 | 5 | 5 | 7 |
| Sorghum_bicolor_20409 | 7 | 4 | 8 | 5 | 7 | 5 | 5 | 7 |
| Triticum_aestivum_4997 | 7 | 4 | 8 | 5 | 7 | 5 | 5 | 7 |
| Triticum_aestivum_5022 | 7 | 4 | 8 | 5 | 7 | 5 | 5 | 7 |
| Zea_mays_1997 | 7 | 4 | 7 | 5 | 7 | 5 | 5 | 7 |
| Zea_mays_1998 | 7 | 4 | 7 | 4 | 7 | 6 | 5 | 7 |
| Zea_mays_2020 | 7 | 4 | 8 | 5 | 5 | 7 | 5 | 7 |
| Tryptophan | | | | | | | | |
| Oryza_nivara_6177 | 7 | 4 | 9 | 5 | 7 | 5 | 5 | 7 |
| Oryza_sativa_3747 | 7 | 4 | 9 | 5 | 7 | 5 | 5 | 7 |
| Saccharum_officinarum_6616 | 7 | 4 | 9 | 5 | 7 | 5 | 5 | 7 |
| Sorghum_bicolor_20433 | 7 | 4 | 9 | 5 | 7 | 5 | 5 | 7 |
| Triticum_aestivum_5043 | 7 | 4 | 9 | 5 | 7 | 5 | 5 | 7 |
| Zea_mays_2041 | 7 | 4 | 9 | 5 | 7 | 5 | 5 | 7 |
| Tyrosine | | | | | | | | |
| Oryza_nivara_6134 | 7 | 4 | 9 | 5 | 7 | 15 | 5 | 7 |
| Oryza_sativa_3702 | 7 | 4 | 9 | 5 | 7 | 14 | 5 | 7 |
| Saccharum_officinarum_6575 | 7 | 4 | 9 | 5 | 7 | 15 | 5 | 7 |
| Sorghum_bicolor_20388 | 7 | 4 | 9 | 5 | 7 | 15 | 5 | 7 |
| Triticum_aestivum_5001 | 7 | 4 | 9 | 5 | 7 | 13 | 5 | 7 |
| Zea_mays_2000 | 7 | 4 | 9 | 5 | 7 | 15 | 5 | 7 |
| Valine | | | | | | | | |
| Oryza_nivara_6160 | 7 | 4 | 9 | 4 | 9 | 6 | 5 | 7 |
| Oryza_nivara_6217 | 7 | 4 | 7 | 5 | 7 | 5 | 5 | 7 |
| Oryza_nivara_6258 | 7 | 4 | 7 | 5 | 7 | 5 | 5 | 7 |
| Oryza_sativa_3782 | 7 | 4 | 7 | 5 | 7 | 5 | 5 | 7 |
| Oryza_sativa_3819 | 7 | 4 | 7 | 5 | 7 | 5 | 5 | 7 |
| Saccharum_officinarum_6601 | 7 | 4 | 9 | 4 | 7 | 6 | 5 | 7 |
| Saccharum_officinarum_6658 | 7 | 4 | 7 | 5 | 7 | 5 | 5 | 7 |
| Saccharum_officinarum_6696 | 7 | 4 | 7 | 5 | 7 | 5 | 5 | 7 |
| Sorghum_bicolor_20464 | 7 | 4 | 7 | 5 | 7 | 5 | 5 | 7 |
| Sorghum_bicolor_20498 | 7 | 4 | 7 | 5 | 7 | 5 | 5 | 7 |
| Triticum_aestivum_5028 | 7 | 4 | 9 | 4 | 7 | 6 | 5 | 7 |
| Triticum_aestivum_5073 | 7 | 4 | 7 | 5 | 7 | 5 | 5 | 7 |
| Triticum_aestivum_5104 | 7 | 4 | 7 | 5 | 7 | 5 | 5 | 7 |
| Zea_mays_2026 | 7 | 4 | 9 | 4 | 7 | 6 | 5 | 7 |
| Zea_mays_2081 | 7 | 4 | 7 | 5 | 7 | 5 | 5 | 79 |
