# Supplementary figures and images for: Genomic and evolutionary aspects of chloroplast tRNA in monocot plants
Source: BMC Plant Biol. 2019 Jan 22;19:39. doi: 10.1186/s12870-018-1625-6 (PMC6341768; doi:10.1186/s12870-018-1625-6)

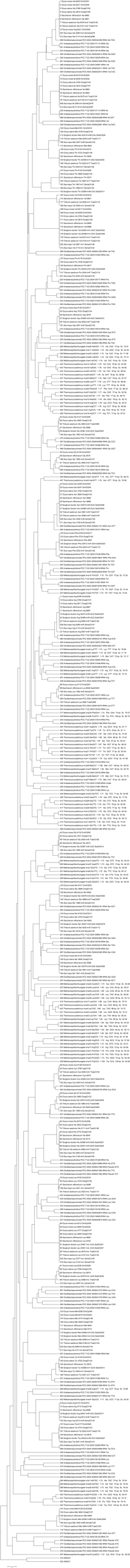

Supplement: Supplementary file 2 — Figure S1. Phylogenetic tree of cyanobacterial tRNAs with tRNAs of Anabaena cyalindrica, Methanococcus maripaludis, Methanospirillum hungatei, Oscillatoria acuminate, and Thermococcus sibiricus. The tRNAs of these species were included as ingroup, whereas, AtNAC1 and AtNAC2 (NAC transcription factor) of Arabidopsis thaliana were used as out-groups. Phylogenetic tree was constructed using the Neighbor-joining method and 1000 bootstrap replicates using MEGA6 software. (PDF 114 kb) [file 12870_2018_1625_MOESM2_ESM.pdf]
